# Composition and Functional Potential of the Human Mammary Microbiota Prior to and Following Breast Tumor Diagnosis

**DOI:** 10.1128/msystems.01489-21

**Published:** 2022-06-01

**Authors:** Courtney Hoskinson, Kelly Zheng, Jaelyn Gabel, Annie Kump, Rana German, Ram Podicheti, Natascia Marino, Leah T. Stiemsma

**Affiliations:** a Natural Science Division, Pepperdine University, Malibu, California, USA; b Susan G. Komen Tissue Bank at the IU Simon Comprehensive Cancer Center, Indianapolis, Indiana, USA; c Center for Genomics and Bioinformatics, Indiana University, Bloomington, Indiana, USA; d Department of Medicine, Indiana University School of Medicine, Indianapolis, Indiana, USA; Institute for Systems Biology

**Keywords:** 16S, breast cancer, breast tissue, functional metagenome, microbiome, transcriptome

## Abstract

Microbiota studies have reported changes in the microbial composition of the breast upon cancer development. However, results are inconsistent and limited to the later phases of cancer development (after diagnosis). We analyzed and compared the resident bacterial taxa of histologically normal breast tissue (healthy, H, *n* = 49) with those of tissues donated prior to (prediagnostic, PD, *n* = 15) and after (adjacent normal, AN, *n* = 49, and tumor, T, *n* = 46) breast cancer diagnosis (*n* total = 159). DNA was isolated from tissue samples and submitted for Illumina MiSeq paired-end sequencing of the V3-V4 region of the 16S gene. To infer bacterial function in breast cancer, we predicted the functional bacteriome from the 16S sequencing data using PICRUSt2. Bacterial compositional analysis revealed an intermediary taxonomic signature in the PD tissue relative to that of the H tissue, represented by shifts in *Bacillaceae*, *Burkholderiaceae*, *Corynebacteriaceae*, *Streptococcaceae*, and *Staphylococcaceae*. This compositional signature was enhanced in the AN and T tissues. We also identified significant metabolic reprogramming of the microbiota of the PD, AN, and T tissue compared with the H tissue. Further, preliminary correlation analysis between host transcriptome profiling and microbial taxa and genes in H and PD tissues identified altered associations between the human host and mammary microbiota in PD tissue compared with H tissue. These findings suggest that compositional shifts in bacterial abundance and metabolic reprogramming of the breast tissue microbiota are early events in breast cancer development that are potentially linked with cancer susceptibility.

**IMPORTANCE** The goal of this study was to determine the role of resident breast tissue bacteria in breast cancer development. We analyzed breast tissue bacteria in healthy breast tissue and breast tissue donated prior to (precancerous) and after (postcancerous) breast cancer diagnosis. Compared to healthy tissue, the precancerous and postcancerous breast tissues demonstrated differences in the amounts of breast tissue bacteria. In addition, breast tissue bacteria exhibit different functions in pre-cancerous and post-cancerous breast tissues relative to healthy tissue. These differences in function are further emphasized by altered associations of the breast tissue bacteria with gene expression in the human host prior to cancer development. Collectively, these analyses identified shifts in bacterial abundance and metabolic function (dysbiosis) prior to breast tumor diagnosis. This dysbiosis may serve as a therapeutic target in breast cancer prevention.

## INTRODUCTION

Approximately 1 in 8 women are diagnosed with breast cancer in their lifetime (1). Breast cancer is also the second leading cause of cancer-related death among women (2). Early detection and diagnosis remain key in improving the prognosis of breast cancer patients. Research focusing specifically on the genetic and environmental factors that influence tumor initiation continues to inform early treatment strategies for this disease (3–5).

In recent years, researchers have begun to elucidate the role of the resident microbiota in the development of breast cancer (6). Specifically, the human mammary microbiota composition is distinguishable between cancerous and healthy breast tissue (7). Compared with tumor tissue, the microbiota composition in tissue adjacent to malignant breast tumors (normal adjacent tissue) also displays a unique bacterial signature, suggesting oncogenic roles for specific bacterial taxa (8). Researchers have also identified variations in the microbiota across breast cancer types (e.g., Human Epidermal Receptor Growth Factor 2-positive, triple-negative, and endocrine-receptor positive breast cancers), and these variations extend beyond the bacterial composition of breast tissue to other resident microbes, such as viruses and fungi (9). Regardless of the microbial species, dysbiosis (microbial imbalance) of the mammary microbiota is consistently correlated with breast tumor development (6). This suggests that an eubiotic microbiota composition is present in healthy breast tissue and plays a role in protecting the breast from tumor initiation and/or progression. To our knowledge, no studies have addressed this question by evaluating the microbiota composition in truly healthy breast tissue. Further, whether dysbiosis occurs at breast tumor initiation and development remains unclear.

Previous studies of the human mammary microbiota used tissue adjacent to benign tumors or tissue from breast reduction or enhancement surgeries as control tissue in comparisons to tumor or adjacent normal tissues (6). However, breast tissue from breast alteration surgeries has significant histological abnormalities compared to tissue voluntarily donated from healthy women (10). The Susan G. Komen Tissue Bank (KTB) at Indiana University Simon Comprehensive Cancer Center (IUSCCC) represents the only repository of truly healthy breast tissue in the world, providing researchers with the unique opportunity to elucidate the genetic, histological, and microbiological characteristics of healthy breast tissue (11). Approximately 5% of KTB tissue donors were later diagnosed with breast cancer (4). These prediagnostic tissues were also included in our study, providing us with the unique opportunity to assess the microbiota in tissue representative of the earliest stages of breast tumor development (4).

In this study, we compared the microbiota of healthy (H) and prediagnostic (PD) breast tissues to that of adjacent normal (AN) and tumor (T) tissues isolated from women diagnosed with breast cancer (*n* = 159). We used 16S rRNA gene sequencing to determine the composition of the bacterial microbiota in these mammary tissues. In addition, we applied the metagenome prediction tool, PICRUSt2, in conjunction with the Kyoto Encyclopedia of Genes and Genomes (KEGG), to predict the functional bacteriome (12, 13). Using these bioinformatic strategies, we were able to identify shifts in bacterial abundance in PD tissue, suggesting that these bacterial shifts preclude the development of breast tumors. Further, our work suggests the breast tissue microbiota is responding to tumor development, as evidenced by the decreased functionality of the bacteriome in PD, AN, and T tissue. Expanding on these findings, we analyzed host-microbiota associations in H and PD tissues and identified altered bacterial associations with the host transcriptome between the two tissue types. Although preliminary, this finding proposes variable interaction of the microbiota with the breast tissue microenvironment prior to cancer diagnosis.

## RESULTS

### Study design and cohort characteristics.

**FIG 1:**
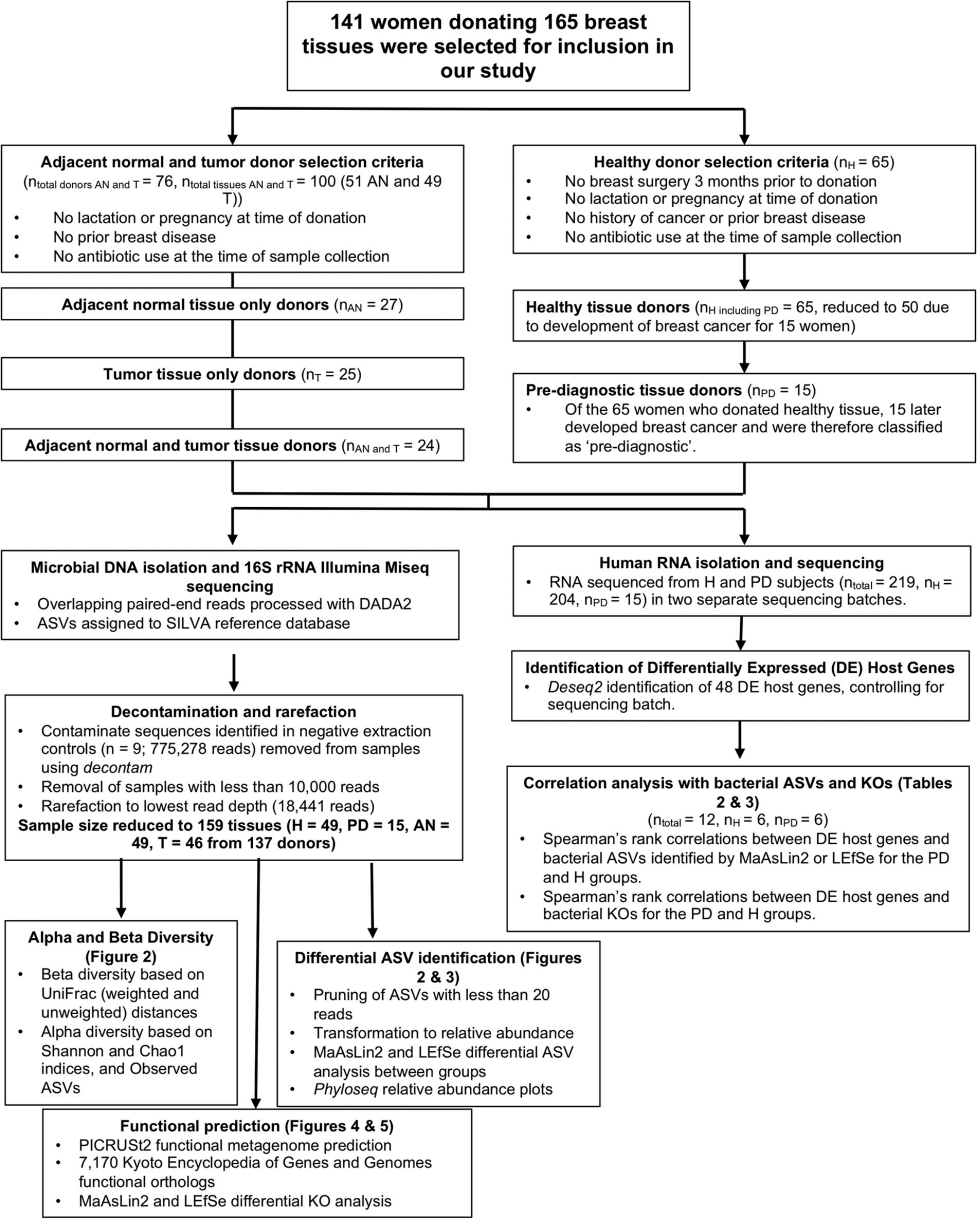
Diagram of donor and sample selection, data preprocessing, and statistical tests performed. A total of 141 female donors were selected for inclusion in our study (76 of whom were diagnosed with breast cancer and donated adjacent normal (AN) or tumor tissue (T) and 65 of whom were healthy at the time of donation (50 donated healthy (H) tissue and 15 donated prediagnostic (PD) tissue). Following the outlined preprocessing methods, the sample size was reduced to 159 tissues (H = 49, PD = 15, AN = 49, and T = 46) from 137 donors.

This project aimed to enhance our mechanistic understanding of the role of the breast tissue microbiota in the development of breast cancer. Breast tissue cores were collected from 141 women, including 65 healthy (H) women and 76 breast cancer patients who donated adjacent normal (AN) and/or tumor (T) tissue (Fig. 1) (11). The healthy cohort included 15 women who were subsequently diagnosed with breast cancer. Therefore, their breast biopsy specimens are classified as prediagnostic (PD) tissue (Fig. 1). The tumor-bearing cohort included 24 women who donated both tumor and adjacent normal tissue and an additional 52 women who donated either AN or T tissue. (Fig. 1). After microbiome sequencing was conducted on all samples from this cohort, sequencing data were pruned according to the parameters outlined in Materials and Methods and the legend to Fig. 1. This step reduced our total cohort size from 141 women (linked with 165 breast tissues) to 137 women, matched with 159 tissues (Fig. 1). A summary of the characteristics of the 137 women included in all microbiome analyses can be found in Table 1.

**TABLE 1:** Cohort characteristics and regression analysis of metadata and cancer status

Variable	*n*, %	*P* value^*a*^	
		H vs cancer groups (AN and T)	H vs PD
Age			
27–45	41, 29.9	0.66	0.36
46–56	45, 32.8	0.49	0.26
57–82	51, 37.2	—	—
Race			
African-American	28, 20.4	0.87	0.98
White	107, 78.1	—	—
NA	2, 1.5	NA	NA
Menopausal status			
Uterine ablation	1, 0.7	NA	0.99
Postmenopausal	53, 38.6	—	—
Premenopausal	32, 23.4	0.048*	0.72
NA	51, 37.2	NA	NA
BMI category			
Normal wt	24, 17.5	—	—
Obese	59, 43.1	0.21	0.79
Overweight	28, 20.4	0.16	0.66
Underweight	2, 1.5	0.99	0.60
NA	24, 17.5	NA	NA
History of cancer			
Yes	1, 0.7	NA	0.99
No	63, 46.0	NA	—
NA	73, 53.3	NA	NA
Status at time of donation			
H	49 (35.46)	NA	NA
PD (tissue collected prior to cancer diagnosis)	15 (10.64)	NA	NA
Diagnosed (has developed breast cancer and donated AN or T tissue)	73 (53.7)	NA	NA

a —, Reference group. NA, not applicable. *, Significant (*P* ≤ 0.05). This analysis only includes individuals in the cohort with available clinical data.

### Compositional overview of the breast tissue microbiota between tissue types.

**FIG 2:**
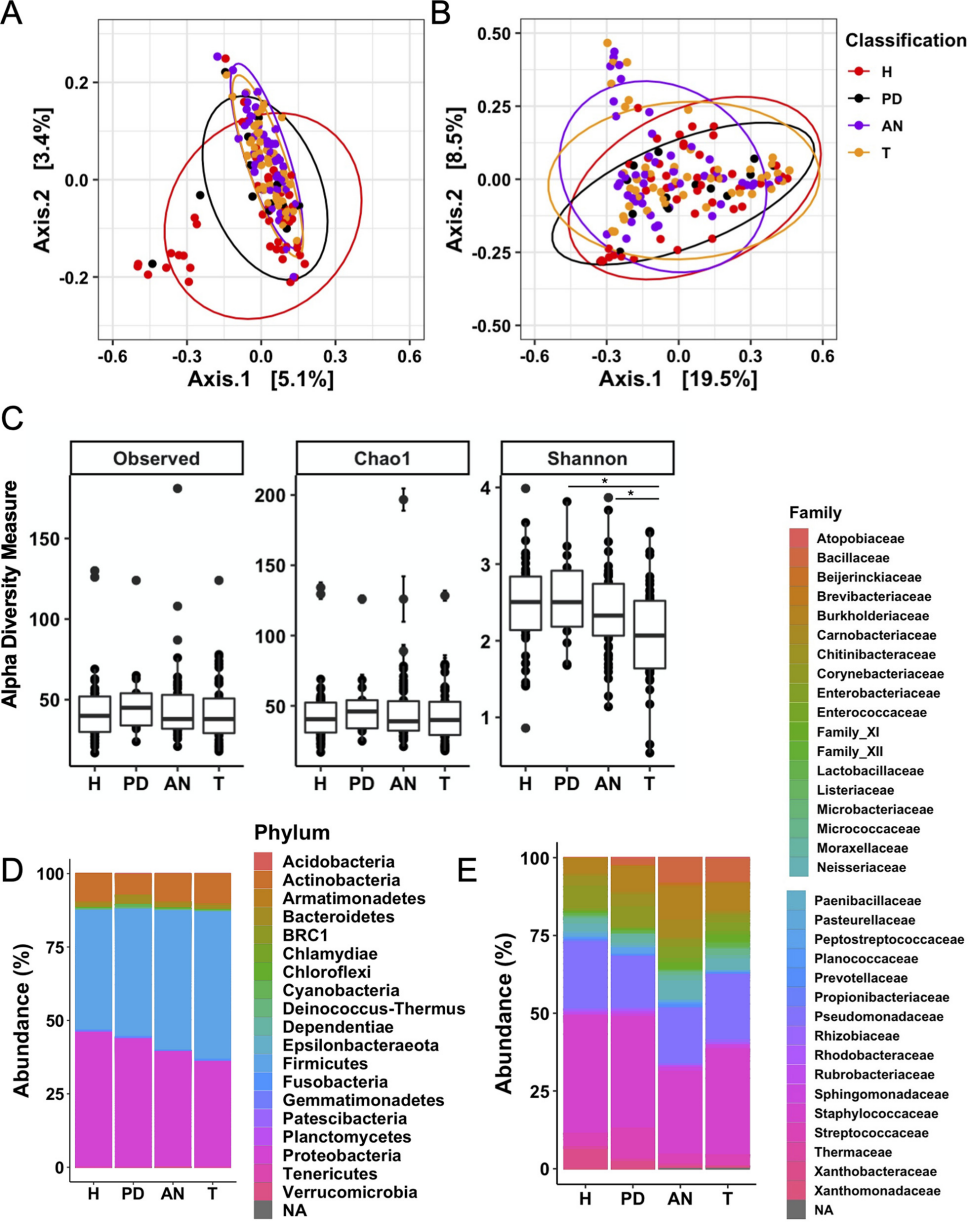
Variations in microbial diversity between H breast tissue, PD breast tissue, and cancerous breast tissues (AN and T). (A and B) Unweighted unique fraction metric (UniFrac) (A) and weighted UniFrac principal coordinates analysis (PCoA) (B) of the mammary microbiota across the four tissue types. (C) Alpha diversity (observed, Chao1, and Shannon diversity index) of the mammary microbiota across the four tissue types. (D) Phylum relative abundance based on all ASVs. (E) Family relative abundances for the top 100 ASVs across the four tissue types. H = 49, PD = 15, AN = 49, T = 46. *, adjusted *P* ≤ 0.05.

Principal coordinate analysis (PCoA) of beta diversity based on weighted and unweighted UniFrac distances showed minor global variations in the composition of the breast tissue microbiota (Fig. 2A and B). These variations are apparent between tissue types, with some low-abundance microbial ASVs potentially contributing to the microbial community structure of some of these tissues (Fig. 2A and B). Because unweighted UniFrac distances consider only the presence or absence of the feature, some H and PD tissues on the left side and some AN tissues on the top portion of the plot appeared to be less similar in community structure to the rest of the tissue samples (Fig. 2A). However, the PCoA based on weighted UniFrac distances takes the abundances of these features into account and diminishes the contribution of low-abundance ASVs (Fig. 2B). When abundances of ASVs were considered, the majority of the tissues (H, PD, AN, and T) overlapped on the PCoA plot. However, segregation between tissues associated with a malignant tumor (AN and T) and tissues collected from healthy women (H and PD) became limited. Betadisper was used to assess community dispersion between tissue types. Although the composition of the tissue types may be similar, there is significant variance/dispersion between tissue communities (*P*_unweighted_ ≤ 0.05 for H-AN, H-T, PD-AN, and PD-T; *P*_weighted_ ≤ 0.05 for H-AN and H-T). Nonmetric multidimensional scaling (NMDS) plots from the weighted and unweighted UniFrac distance measures revealed enhanced variability in the AN and T tissues compared to the H and PD tissues (see Fig. S1 in the supplemental material). This study was not designed to address differences in the breast tissue microbiota among breast tumor types (i.e., histologic subtype, estrogen receptor positivity, etc.), but perhaps the variability noted in the cancerous tissues (AN and T) is an artifact of the type of malignant tumor diagnosed.

10.1128/msystems.01489-21.1 FIG S1 NMDS plots based on unweighted (A) and weighted (B) UniFrac distances. Download FIG S1, PDF file, 0.2 MB. Copyright © 2022 Hoskinson et al. 2022 Hoskinson et al. https://creativecommons.org/licenses/by/4.0/ This content is distributed under the terms of the Creative Commons Attribution 4.0 International license.

We compared alpha diversity among tissue types based on observed ASVs (richness) and the Chao1 and Shannon diversity indices (Fig. 2C). There were no significant differences in alpha diversity based on observed ASVs or the Chao1 index. However, when the distribution of ASVs is considered, we identified significant differences between H and T and PD and T tissues (*P ≤ *0.05). This finding corroborates the PCoA and NMDS analysis, suggesting that enhanced variability in the tumor microenvironment is affecting microbiota composition.

A comparison of the aggregated ASVs at the phylum level (Fig. 2D) revealed that, regardless of the tissue type, three phyla, *Proteobacteria*, *Firmicutes*, and *Actinobacteria*, dominate the breast tissue. Analysis of aggregated ASVs in the top 100 bacterial families (Fig. 2E) showed distinct compositional variations, which suggested a unique bacterial compositional signature present in tissue prior to and after tumor development. Specifically, families such as *Bacillaceae* and *Burkholderiaceae* increased in abundance in breast tissue isolated from women who developed breast cancer (PD, AN, and T) compared with those from healthy women (Fig. 2E). Conversely, the family *Xanthobacteraceae* decreased in abundance in PD, AN, and T tissues compared with H tissues. As should be expected, individual abundance profiles at the phylum and family levels among the four tissue types (Fig. S2 and S3) highlighted interindividual variability. Phylum-level analysis indicated two common breast tissue microbiota profiles irrespective of cancer status or tissue type. One profile was characterized by higher *Proteobacteria*, whereas in the other, *Firmicutes* were more dominant. Following this analysis, we addressed taxon-specific contributions to these global variations in microbiota diversity between tissue types.

10.1128/msystems.01489-21.2 FIG S2 Individual profiles of relative phylum abundance in H tissues (A), PD tissues (B), AN tissues (C), and T tissues (D). Download FIG S2, PDF file, 0.4 MB. Copyright © 2022 Hoskinson et al. 2022 Hoskinson et al. https://creativecommons.org/licenses/by/4.0/ This content is distributed under the terms of the Creative Commons Attribution 4.0 International license.

10.1128/msystems.01489-21.3 FIG S3 Individual profiles of relative family abundance in H tissues (A), PD tissues (B), AN tissues (C), and T tissues (D). Download FIG S3, PDF file, 0.3 MB. Copyright © 2022 Hoskinson et al. 2022 Hoskinson et al. https://creativecommons.org/licenses/by/4.0/ This content is distributed under the terms of the Creative Commons Attribution 4.0 International license.

### Analysis of differentially abundant taxa in PD, AN, and T tissue relative to H tissue.

**FIG 3:**
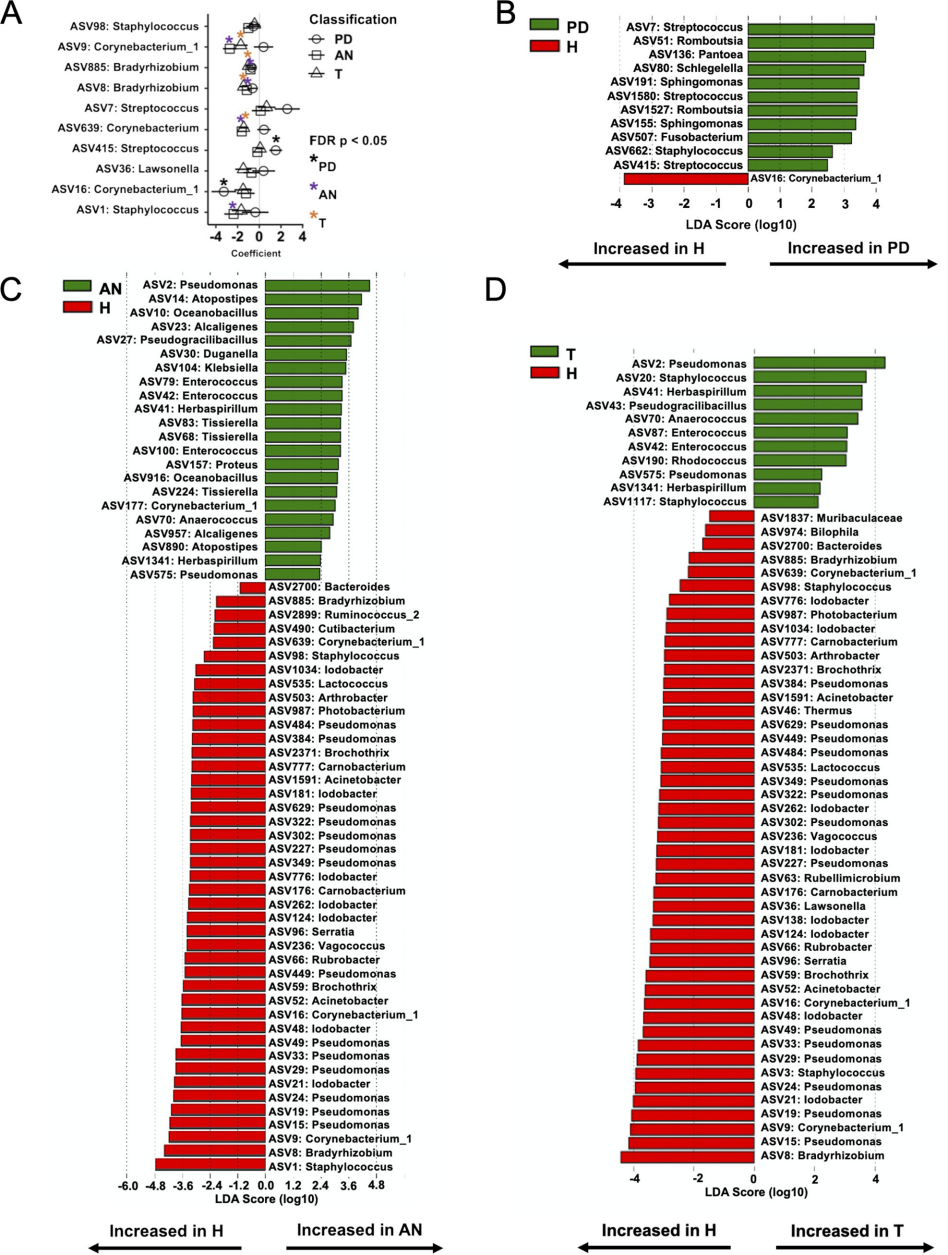
Differentially abundant taxa in human mammary tissue subtypes. (A) MaAsLin2 analysis of differential ASV abundance in tissue subsets with H tissue as reference. Counts were transformed to relative abundance prior to analysis. Taxa shown have a *q* value (Benjamini-Hochberg adjustment) cutoff of 0.25. *, *P* ≤ 0.05. Shapes correspond to tissue subsets (circle, PD; square, AN; triangle, T). (B to D) LEfSe analysis for H versus PD (B), AN (C), and T (D). One-against-all analyses and default settings were applied using the LEfSe Galaxy platform. Taxa shown have a *P* value cutoff of 0.05.

We used MaAsLin2 and Linear discriminant analysis Effect Size (LEfSe) to identify differentially abundant bacterial taxa in PD, AN, and T tissue relative to the H tissue (Fig. 3 and Fig. S4 and Table S1). MaAsLin2 identified 10 ASVs as differentially abundant in the PD, AN, or T tissues relative to H tissue (*q *≤ 0.25) (Fig. 3A). Among these, four ASVs were associated with PD tissue (*q *≤ 0.25, 7, 16, 415, and 885) (Fig. 3A). LEfSe analysis corroborated the MaAsLin2 findings in PD tissue, identifying ASVs 7, 16, 415, and 885 as differentialy abundant in PD tissue relative to H tissue (alpha ≤ 0.05) (Fig. 3B). ASVs 7 and 415 represented Streptococcus spp., which appeared to be uniquely increased in PD tissue compared with the other three tissues (Fig. 3B). ASV 16 classified to *Corynebacterium 1* and 885 to *Bradyrhizobium* spp. Interestingly, AN and T tissues followed similar trends of decreased abundance of these features relative to the H tissue (nonsignificant from MaAsLin2, ASVs 9 and 16, *Corynebacterium 1*, and 885, *Bradyrhizobium* spp., identified as more abundant in H tissue than AN and T via LEfSe, alpha ≤ 0.05) (Fig. 3A, C, and D). MaAsLin2 identified Staphylococcus spp. (ASVs 1 and 98) as differentially abundant in AN tissue (*q *≤ 0.25), with similar trends of decreased abundance (not significant) in PD and T tissue relative to H tissue (Fig. 3A). LEfSe supported these findings by identifying an increase in Staphylococcus spp. in H tissue relative to AN (ASVs 1 and 98) and T (ASVs 3 and 98) tissues (Fig. 3C and D). Aside from these similar trends, LEfSe analysis also revealed increased abundance of ASVs 191 and 155 (classified as *Sphingomonas* spp.) in PD tissue relative to H tissue (Fig. 3B). In addition, a number of ASVs classified as Pseudomonas spp. and *Lodobacter* spp. were increased in H tissue relative to AN and T tissues (alpha ≤ 0.05) (Fig. 3C and D). Conversely, ASVs classified as *Enterococcus* spp. were increased in AN and T tissues relative to H tissue (alpha ≤ 0.05) (Fig. 3C and D). Collectively, these variations in bacterial abundance suggest the presence of a unique bacterial signature prior to tumor development (PD tissue) and possibly persistence and enhancement of this signature following tumor development (AN and T tissues). Further, both MaAsLin2 and LEfSe identified more ASVs decreased in abundance in PD, AN, and T tissue than H tissue (as opposed to increased), suggesting response of the breast tissue microbiota to tumor development (Fig. 3A to D).

10.1128/msystems.01489-21.4 FIG S4 Scatterplots comparing the relative abundances of taxa and KOs identified with MaAsLin2 and/or LEfSe. Download FIG S4, PDF file, 2.8 MB. Copyright © 2022 Hoskinson et al. 2022 Hoskinson et al. https://creativecommons.org/licenses/by/4.0/ This content is distributed under the terms of the Creative Commons Attribution 4.0 International license.

10.1128/msystems.01489-21.8 TABLE S1 All taxa and KOs identified by MaAsLin2 TABLE S1, XLS file, 2.8 MB. Copyright © 2022 Hoskinson et al. 2022 Hoskinson et al. https://creativecommons.org/licenses/by/4.0/ This content is distributed under the terms of the Creative Commons Attribution 4.0 International license.

### Analysis of the functional bacteriome highlights decreased abundance of metabolic pathways prior to and after breast cancer diagnosis.

**FIG 4:**
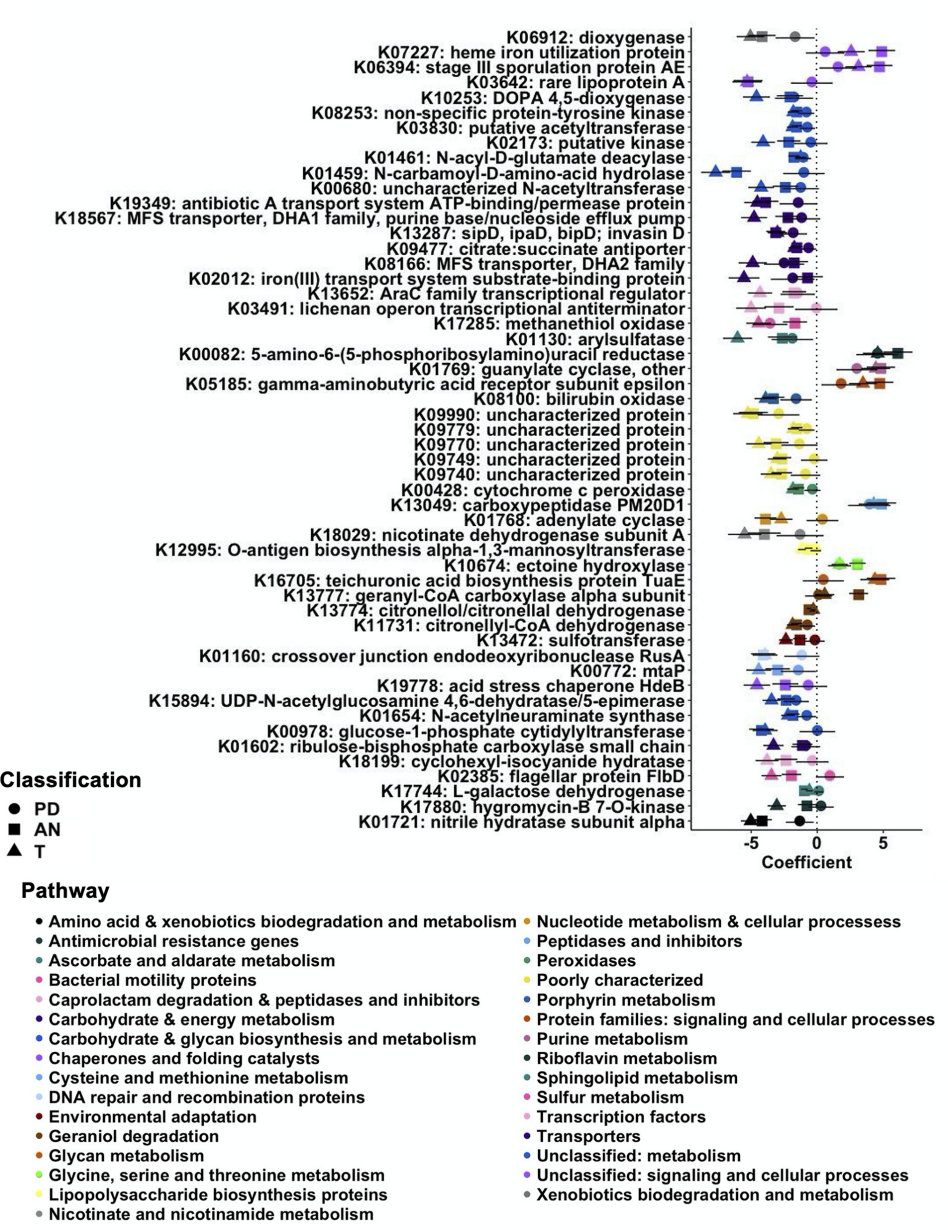
Differential abundances of bacterial functional pathways in human mammary tissue subtypes. MaAsLin2 differential analysis of KEGG ortholog abundance in tissue subsets with H tissue as reference. Counts were transformed to relative abundance prior to analysis. The top 50 most significant KEGG orthologs ranked by lowest *q* value are shown with all group MaAsLin2 coefficients included. All KEGGs shown have an adjusted *q* value (Benjamini-Hochberg adjustment) cutoff of 0.01 for at least one tissue subset. Shapes correspond to tissue subsets (circle, PD; square, AN; triangle, T), while colors correspond to higher-level KEGG pathway classifications (reverse alphabetical order).

**FIG 5:**
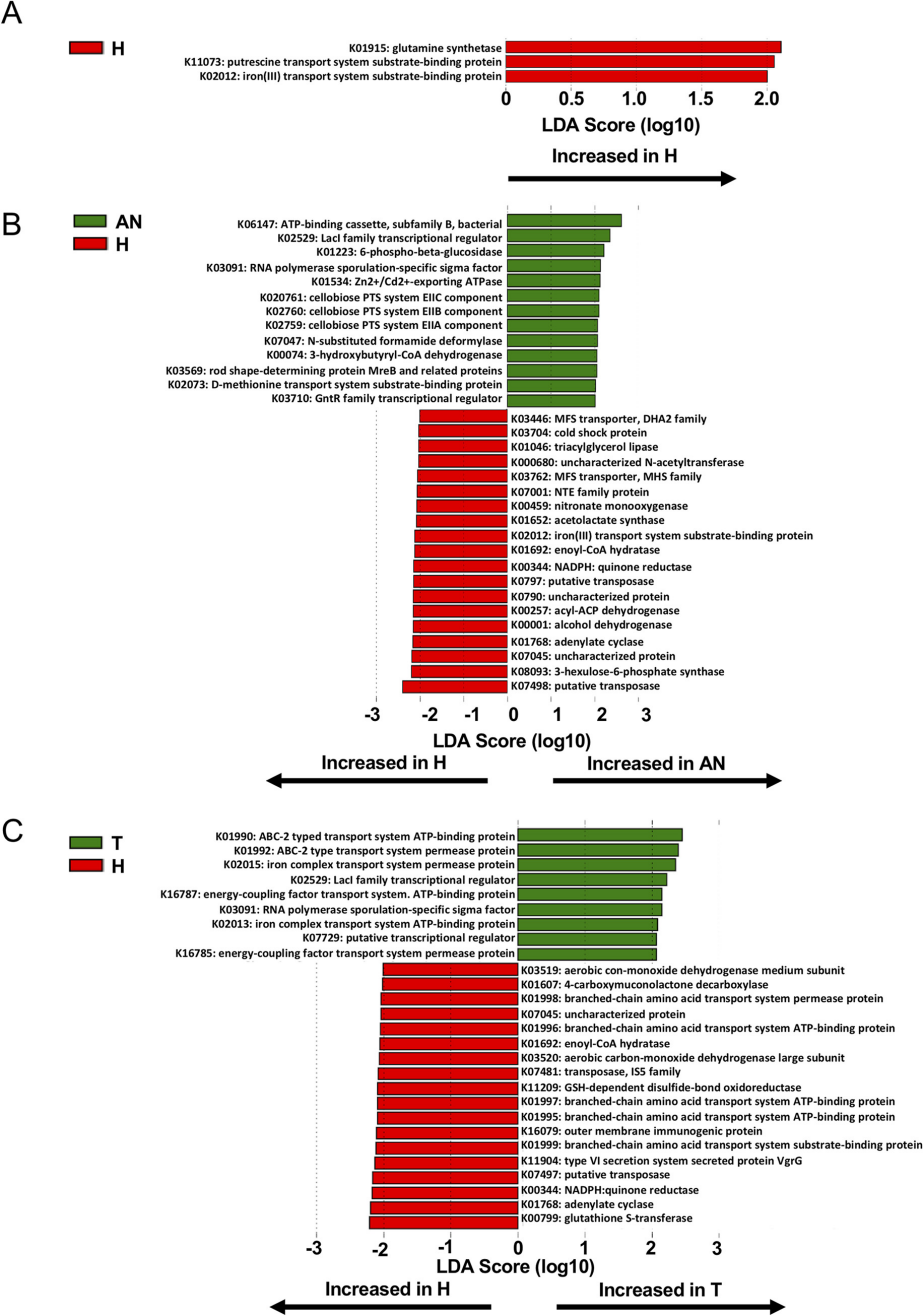
Differential abundance analysis of KEGG orthologs in human mammary tissue subtypes. (A to C) LEfSe for H versus PD (A), AN (B), and T (C). One-against-all analyses and default settings were applied using the LEfSe Galaxy platform. Taxa shown have a *P* value cutoff of 0.05.

We used PICRUSt2 (14) and the KEGG database (12) to predict the bacterial functional metagenome from the 16S amplicon sequencing data. The analysis identified 7,170 KEGG orthologs (KO), otherwise referred to as nodes or steps in KEGG pathway maps, correlated with our 16S amplicon sequencing data. We used MaAsLin2 and LEfSe to determine differentially abundant microbial KOs in PD, AN, and T tissue relative to H tissue (Fig. 4). MaAsLin2 identified 574 KOs as significantly associated (*q* ≤ 0.05; Table S1) with PD, AN, or T tissue. Of these KOs, 395 are less abundant in PD, AN, or T tissue relative to H tissue. Figure 4 shows the top 50 ranked KOs based on *q* value. This figure includes coefficients for PD, AN, and T tissue for the top 50 KOs to emphasize the reduction in bacterial function trending among all tissues from women who had cancer at donation (AN and T) or those who developed cancer after donation (PD) (significant and non-significant; see Table S1 for associated *q* values per group). LEfSe was used for a between-group analysis (PD-H, AN-H, and T-H) of KOs and supported the MaAsLin2 findings (Fig. 5). Specifically, LEfSe identified three KOs in the PD-H comparison, and all were increased in H tissue relative to PD tissue (Fig. 5). In addition, 19 KOs were increased in H relative to AN tissue (compared with 13 increased in AN tissue), and 18 KOs were increased in H relative to T tissue (compared with nine increased in T tissue) (Fig. 5). The majority of these pathways were linked to aspects of bacterial metabolism or nutrient transport, suggesting a decreased metabolic response on the microbiota prior to (PD) and after development of (AN and T) breast tumors.

### Associations between the host transcriptome and the breast microbiota are altered in PD tissues relative to H tissues.

To determine how the mammary microbiota and its compositional and functional changes in early cancer development affected the local tissue, we conducted Spearman’s rank correlation analyses between host transcriptome profiles, bacterial ASVs, and functional KOs for a subset of subjects in the PD and H groups (total *n*  = 12, *n*_H_ = 6, *n*_PD_ = 6) (Table S2). For the correlation analysis between the host transcriptome and bacterial taxa, we identified no statistically significant correlations after *P* value adjustment in the H group and 2 correlations (*P* adjusted ≤ 0.05) in the PD group (Tables 2 and 3 and Fig. S5). Spearman’s rank correlation analysis of bacterial KOs and the host transcriptome identified 41 statistically significant (*P* adjusted ≤ 0.05) associations between KOs and host genes in the H group and 13 statistically significant (*P* adjusted ≤ 0.05) associations between KOs and host genes in the PD group. Of note, in the H group *CYP24A1* (encoding the enzyme 24-hydroxylase) was inversely associated with a number of bacterial KOs within the nutrient transport and metabolic pathways. Interestingly, this same gene was positively associated with the bacteriome in PD tissue (two KOs classified as microbial metabolism in diverse environments and cell signaling). It is also interesting that the majority of the KO-gene correlations in PD tissue were positive (12 out of 13), whereas the majority of KO-gene correlations in H tissue included inverse correlations (39 out of 41). Although these findings are not indicative of a causal impact of the microbiota in breast tumor development or a response of these bacteria to breast tumor development, they support our taxonomic and functional assessments of the breast tissue microbiota. The analysis also highlights possible differences in the interaction of the breast tissue microbiota with the tissue microenvironment in healthy women and in women at the preliminary stages of breast tumor development.

**TABLE 2:** Correlations between genes and taxa

Classification	ASV	Ensembl gene ID	Gene name or description	*R*	Adjusted *P* value
Healthy	No significant correlations				
Prediagnostic	ASV191, *Sphingomonas*	ENSG00000212663	*Novel transcript*	1	0
	ASV1580, Streptococcus	ENSG00000267676	*THA1P*	1	0

**TABLE 3:** Correlations between genes and KEGG orthologs

Group and KO no.	Pathway	Ensembl gene ID	Gene name or description	*R*	Adjusted *P* value
Healthy					
K01150	Enzymes	ENSG00000019186	*CYP24A1*	−1	0
K01545	Two-component system	ENSG00000019186	*CYP24A1*	−1	0
K01795	Fructose and mannose metabolism	ENSG00000019186	*CYP24A1*	−1	0
K03314	Transporters	ENSG00000019186	*CYP24A1*	−1	0
K03668	Genetic information processing	ENSG00000019186	*CYP24A1*	−1	0
K04337	Secretion system	ENSG00000019186	*CYP24A1*	−1	0
K04338	Secretion system	ENSG00000019186	*CYP24A1*	−1	0
K06887	Function unknown	ENSG00000019186	*CYP24A1*	−1	0
K07338	Function unknown	ENSG00000019186	*CYP24A1*	−1	0
K07351	Bacterial motility proteins	ENSG00000019186	*CYP24A1*	−1	0
K09932	Function unknown	ENSG00000019186	*CYP24A1*	−1	0
K10025	ABC transporters	ENSG00000019186	*CYP24A1*	−1	0
K10844	Genetic information processing	ENSG00000019186	*CYP24A1*	−1	0
K11016	Bacterial secretion system	ENSG00000019186	*CYP24A1*	−1	0
K11017	Bacterial secretion system	ENSG00000019186	*CYP24A1*	−1	0
K11383	Two-component system	ENSG00000019186	*CYP24A1*	−1	0
K11477	Poorly characterized	ENSG00000019186	*CYP24A1*	−1	0
K11739	Poorly characterized	ENSG00000019186	*CYP24A1*	−1	0
K12253	Amino acid metabolism	ENSG00000019186	*CYP24A1*	−1	0
K12981	Lipopolysaccharide biosynthesis	ENSG00000019186	*CYP24A1*	−1	0
K14744	Unclassified: metabolism	ENSG00000019186	*CYP24A1*	−1	0
K15650	Polyketide biosynthesis proteins	ENSG00000019186	*CYP24A1*	−1	0
K15737	Microbial metabolism in diverse environments	ENSG00000019186	*CYP24A1*	−1	0
K16517	Transporters	ENSG00000019186	*CYP24A1*	−1	0
K18093	Two-component system	ENSG00000019186	*CYP24A1*	−1	0
K18294	Genetic information processing	ENSG00000019186	*CYP24A1*	−1	0
K18540	Unclassified: metabolism	ENSG00000019186	*CYP24A1*	−1	0
K19155	Prokaryotic defense system	ENSG00000019186	*CYP24A1*	−1	0
K19609	Two-component system	ENSG00000019186	*CYP24A1*	−1	0
K19610	Two-component system	ENSG00000019186	*CYP24A1*	−1	0
K12266	Genetic information processing	ENSG00000130741	*EIF2S3*	−1	0
K12549	Signaling and cellular processes	ENSG00000130741	*EIF2S3*	−1	0
K02673	Secretion system	ENSG00000134245	*WNT2B*	−1	0
K07215	Biosynthesis of secondary metabolites	ENSG00000134245	*WNT2B*	−1	0
K07481	Genetic information processing	ENSG00000134245	*WNT2B*	−1	0
K00032	Microbial metabolism in diverse environments	ENSG00000226747	*FSIP2-AS2*	−1	0
K11441	Microbial metabolism in diverse environments	ENSG00000226747	*FSIP2-AS2*	−1	0
K12256	Metabolic pathways	ENSG00000226747	*FSIP2-AS2*	−1	0
K06214	Secretion system	ENSG00000246334	*PRR7-AS1*	−1	0
K00467	Carbohydrate metabolism	ENSG00000267676	*THA1P*	1	0
K14470	Microbial metabolism in diverse environments	ENSG00000267676	*THA1P*	1	0
Prediagnostic					
K02011	ABC transporters	ENSG00000185551	*NR2F2*	1	0
K06970	Genetic information processing	ENSG00000185551	*NR2F2*	1	0
K01027	Carbohydrate metabolism	ENSG00000212663	*Novel transcript*	1	0
K01387	Peptidases and inhibitors	ENSG00000212663	*Novel transcript*	1	0
K07647	Two-component system	ENSG00000212663	*Novel transcript*	1	0
K10020	ABC transporters	ENSG00000212663	*Novel transcript*	1	0
K06320	Signaling and cellular processes	ENSG00000228782	*MRPL45P2*	1	0
K07215	Biosynthesis of secondary metabolites	ENSG00000243678	*NME2*	−1	0
K06407	Signaling and cellular processes	ENSG00000268460	*LOC93429*	1	2.17E−28
K00387	Microbial metabolism in diverse environments	ENSG00000019186	*CYP24A1*	1	8.69E−28
K19336	Signaling and cellular processes	ENSG00000019186	*CYP24A1*	1	8.69E−28
K00467	Carbohydrate metabolism	ENSG00000211639	*IGLV4-60*	1	8.69E−28
K14470	Microbial metabolism in diverse environments	ENSG00000211639	*IGLV4-60*	1	8.69E−28

10.1128/msystems.01489-21.5 FIG S5 Scatterplots of significant taxon-gene or KO-gene correlations. Download FIG S5, PDF file, 0.7 MB. Copyright © 2022 Hoskinson et al. 2022 Hoskinson et al. https://creativecommons.org/licenses/by/4.0/ This content is distributed under the terms of the Creative Commons Attribution 4.0 International license.

10.1128/msystems.01489-21.9 TABLE S2 Data used for correlation analyses TABLE S2, XLS file, 0.1 MB. Copyright © 2022 Hoskinson et al. 2022 Hoskinson et al. https://creativecommons.org/licenses/by/4.0/ This content is distributed under the terms of the Creative Commons Attribution 4.0 International license.

## DISCUSSION

Current studies suggest that differences in bacterial composition in human mammary tissue are associated with breast cancer (6). However, to our knowledge, no research has addressed the role of the breast tissue microbiota in the earliest stages of breast tumor development. We undertook this task by analyzing histologically normal breast tissues donated prior to the clinical diagnosis of breast cancer (PD). Previous microbiota studies have also compared breast tumor tissue to adjacent normal and/or breast tissue isolated from breast augmentation surgery, both of which possess significant histological and immunological abnormalities compared to tissue donated from healthy women (4, 10). Further, few of the current compositional analyses of the human mammary microbiota have been conducted in combination with a global functional assessment of the resident bacteria. Our study addressed each of these gaps in the current literature. We determined the bacterial composition of healthy (H) (*n* = 49), prediagnostic (PD) (*n* = 15), adjacent normal (AN) (*n* = 49), and tumor (T) (*n* = 46) breast tissues. Through this analysis, we identified bacterial dysbiosis prior to the onset of breast cancer (PD tissue), which we found to be enhanced following breast tumor development (AN and T tissues). We also predicted the functional bacteriome from the 16S amplicon sequencing data and identified significant metabolic dysregulation associated with this bacterial dysbiosis in PD, AN, and T tissues relative to H breast tissue. Lastly, we analyzed correlations between the host transcriptome, microbial taxa, and functional KOs and identified altered correlative patterns between the microbiota and host transcriptome when comparing PD tissue to H tissue.

A truly healthy breast tissue microbiota has not yet been characterized. In our cohort, the H breast tissue microbiota is represented by three major phyla, *Proteobacteria*, *Firmicutes*, and *Actinobacteria* (Fig. 2D and Fig. S3A) (7, 15), yet there is clear interindividual variability even at the high phylum and family taxonomic ranks (Fig. S2A and S3A). Consistent with most current studies of the human mammary microbiota, we report a decrease in bacterial diversity (*P ≤ *0.05 based on the Shannon index, not significant based on observed ASVs or Chao1) in T tissue relative to H and PD tissue (Fig. 2B) (6). Relative abundance analysis and analysis of differentially abundant taxa using MaAsLin2 and LEfSe identified significant taxonomic variation in the bacterial microbiota between the four tissue types (Fig. 2 and 3). Specifically, we identified an increased abundance of *Bacillaceae* and Streptococcus spp. in tissues from women who developed cancer (PD, AN, and T), which is also consistent with previous studies (9, 15).

Along with our novel characterization of the microbiota in H breast tissue, our study is the first to characterize the breast microbiota in tissue prior to breast cancer diagnosis (PD tissue). The PD tissue microbiota was most similar to the microbiota composition of H breast tissue (Fig. 2 and 3 and Fig. S2B and S3B). However, the PD tissue microbiota appeared as an intermediate compositional signature, indicative of the beginning of dysbiosis in the breast prior to breast tumor development. Relative to the H tissue microbiota composition, there were several bacterial taxa that followed trends in abundance similar to those found in AN and T tissue (e.g., *Bacillaceae*, *Burkholderiaceae*, *Corynebacteriaceae*, *Enterobacteriaceae*, *Xanthobacteriaceae*, *Staphylococcaceae*) (Fig. 2 and 3 and Fig. S2 and S3). PD breast tissue displayed a phylum profile similar to that of microbiotas of patients at high risk for breast cancer reported by Tzeng et al., although these profiles differed at lower taxonomic ranks (16). We also identified taxa in PD tissue that were previously reported as differentially abundant in cancerous tissues. Specifically, studies report a higher abundance of Streptococcus and *Corynebacterium 1* in cancerous tissue relative to control tissue (9, 16). Urbaniak et al. report decreased abundance of *Bacillus* and Staphylococcus in cancerous tissue relative to healthy controls (7). These findings are consistent with our comparisons of PD, AN, and T tissue microbiotas to the H microbiota. Thus, although the PD tissue microbiota is similar in composition to that of H tissue, there are clear shifts in bacterial abundance that preclude breast tumor diagnosis, and these shifts are also present in breast tissue associated with malignant tumors (AN and T tissues).

We used PICRUSt2 to predict the functional bacteriome based on our 16S amplicon sequencing data. The majority of genes identified are related to the metabolic capacity of the microbiota (Fig. 4 and 5). Some of these metabolic pathways suggest decreased bacterial function, which would otherwise be protective against breast cancer development. For example, we identified a decreased abundance of bacterial genes associated with xenobiotics degradation. Degradation of carcinogenic xenobiotics into nontoxic bioproducts is shown to be protective against breast carcinogenesis (17). We also note an increased abundance of KOs associated with bacterial pathogenesis and defense. Specifically, K13734 (fibronectin binding-protein/bacterial invasion of epithelial cells) and K07464 (CRISPR-cas4) correspond to bacterial pathogenic and defense functions. Although the differentially abundant ASVs identified in the PD group are only classified down to the genus level, Streptococcus spp. are common pathogens of the human breast (18). Presence of these bacteria prior to tumor development might instigate an inflammatory response in breast tissue or a tumor-inducing microenvironment. Beyond these specific functional anomalies, our analysis of microbiota function in relation to breast cancer highlights significant metabolic reprogramming in the resident microbiota. Notably, we observed the reduction of glutathione (GSH) metabolism (K00383, K00432, and K00799) in AN and T tissue compared with H tissue (Table S1). GSH metabolism plays a critical role in cancer initiation, as it mediates the removal and detoxification of carcinogens (19). Alterations in this pathway can affect cell survival and promote tumor progression. Metabolic reprogramming and alterations in cell activity are also emerging hallmarks of a variety of cancers, including breast cancer (20). Marino et al. recently conducted an analysis of the host-transcriptome among similar prediagnostic tissues collected by the KTB. In this study, lipid metabolism genes were upregulated prior to breast tumor development (4). Here, we show an underrepresentation of bacterial lipid metabolism genes in PD and AN breast tissue (Fig. 4A and 5B and Table S1). Given these findings, it is possible that the precancerous human cells are exhibiting Warburg metabolism, leading to their inevitable overproliferation and enhanced metabolic capacity (21). This may be negatively affecting the resident microbiota of mammary tissue, resulting in decreased microbial metabolism and enhanced metabolic dysregulation in response to uncontrollable growth and utilization of metabolites by host-cancerous tissue.

To investigate the cross talk between the microbiota and the local microenvironment, we conducted a Spearman’s rank correlation analysis between the host transcriptome and microbial taxa and genes. We observed inverse host-microbial correlative patterns among a subset of PD and H tissues (Tables 2 and 3). Specifically, the majority of correlations in PD tissue between taxa and the host transcriptome and microbial KOs and the host transcriptome were positive, while in H tissue, the majority of microbial KO-host transcriptome correlations are negative, noting an inverse relationship between microbial function and host gene expression. Of note, the *CYP24A1* gene, which encodes the enzyme 24-hydroxylase, was among the host genes inversely associated with microbial genes in H tissue and positively associated with microbial genes in PD tissue. This enzyme is in the cytochrome P450 family of enzymes, which are involved in steroid hormone and xenobiotics metabolism (22). There is evidence of increased expression of cytochrome p40 genes in breast cancer (22). It is possible we are seeing a correlated bacterial response in PD tissue to a changing tissue microenvironment in the earliest stages of breast tumor development. Aside from the correlations with *CYP24A1*, nucleoside diphosphate kinase 2, *NME2*, is a metastasis suppressor in many types of cancer (23). Our data suggest that in PD tissue, as the function of certain bacteria increases, expression of *NEM2* diminishes, highlighting a potential mechanistic connection between the breast tissue microbiota (specifically taxa that are increased in abundance in PD tissue) and the tumor microenvironment. The functional KOs associated with the host transcriptome include metabolic pathways, the secretion system, and lipopolysaccharide biosynthesis, suggesting a diverse array of functional associations between the resident bacteriome and host transcriptome. Together with our compositional and functional data, these findings also highlight possible response pathways of the resident mammary bacteria to breast cancer development.

A significant strength of our study is our characterization of the human mammary microbiota composition and functional potential prior to cancer development, which has not been previously conducted. To conduct this analysis, we used a nested PCR to enrich bacterial sequences in these low-biomass breast tissue samples. This nested library preparation has been suggested to enhance the ability to interpret microbiota variations in low-biomass samples (24). Additionally, the H and PD breast tissues used in this study are precious and limited in availability. PICRUSt2’s metagenome prediction is highly comparable to shotgun metagenomic analysis of the microbiota (14), maximizing the utilization of these tissues for a variety of future genomic analyses.

Our study is not without limitations. The PD tissue subset is small (*n* = 15), which limits our statistical power to identify variation in the PD microbiota. We are working with the KTB to identify PD donors and increase the sample cohort. Moreover, our identification of a PD microbial compositional and functional signature, though novel, does not clarify whether the breast tissue-resident bacteria are causally implicated in breast tumor development.

Although many questions related to causality remain, our work highlights yet another role of the resident microbiota in human disease. Through this analysis of the human mammary microbiota, we were able to identify, for the first time, a unique microbial compositional signature that precludes the development of breast tumors (PD tissue). We identified significant metabolic dysregulation of the microbiota in tissues from women who developed (PD tissue) or were currently diagnosed with breast cancer (AN and T). We also identified altered correlative patterns of the microbiota with the host transcriptome in PD tissue compared to H tissue. An expansion on this analysis of host-microbiota interactions in a larger sample size of PD tissues represents a future step to further elucidate how these microbes promote breast cancer and how they could be harnessed to potentially protect against this disease.

## MATERIALS AND METHODS

### Breast tissue sample procurement.

A total of 165 fresh-frozen breast tissue samples were obtained from the Indiana University Simon Comprehensive Cancer Center Tumor Bank (IUSCCC) and the Susan G. Komen Tissue Bank at IUSCCC (KTB) for microbiota analysis under protocols approved by the Indiana University Institutional Review Board (IRB; protocol number 1011003097 and protocol number 11438, respectively). Samples from both banks are stored and managed by the Biospecimen Collection and Banking Core. The samples were grouped into the following categories: prediagnostic (PD; *n* = 15), healthy (H; *n* = 50), adjacent normal (AN; *n* = 51; postdiagnosis adjacent tissue, 5 cm, adjacent to breast tumors), and tumor (T; *n* = 49) tissues. Tissue from healthy women was selected from the KTB, a unique repository of voluntarily donated healthy breast tissue, available to researchers as control tissue in studies aimed at understanding the molecular and histological traits involved in breast cancer development (11). In addition, among the women who donated healthy breast tissue to the KTB, we identified 15 women who donated healthy tissue but were later, unfortunately, diagnosed with breast cancer (PD). AN and T tissues were obtained from the IUSCCC tumor bank. The following breast biopsy procedure performed by the KTB is described on their website (https://komentissuebank.iu.edu/researchers/sop.php). Briefly, donors complete informed consent paperwork, are measured for height and weight, and fill out an online questionnaire, thereby self-reporting clinical data (age, menopausal status, etc.) during sample procurement. The mammary skin is sterilized and numbed with 10 ml of 1% lidocaine. A nick incision is made with a sterile scalpel, and up to six cores are taken from the upper outer quadrant of the breast using the ATEC breast biopsy system (Hologic Inc., Bedford, MA). The tissue cores are then transported to the tissue processing room and flash-frozen in liquid nitrogen within 10 min. Samples are subsequently stored at –195°C until being shipped to corresponding labs for study.

### Subject selection.

For the prediagnostic cohort (PD), tissue cores donated by 15 women were selected for microbiota analysis based on sample availability. For the healthy subset (H), we requested samples from women meeting the following criteria: healthy individuals with no prior breast surgery within 3 months of donating, no lactation or pregnancy at the time of sample collection, no personal history of cancer or benign breast disease, and no antibiotic use at the time of sample collection. For the women with cancer, we selected adjacent normal (AN) and tumor (T) tissue from women meeting the following criteria (prioritizing women who donated both tumor and adjacent normal tissue): no lactation or pregnancy at the time of sample collection, no prior breast disease (where possible), and no antibiotic use at the time of sample collection. Based on these criteria, our cohort consisted of 141 women: 76 women donated either T tissue (*n* = 25), AN tissue (*n* = 27), or both (*n* = 24), 50 women donated H tissue, and 15 women donated PD tissue. In addition, subsequent to screening based upon respective health histories, 6 samples containing fewer than 10,000 sequencing reads were removed from further study (1 H sample, 2 AN samples, and 3 T samples) to avoid inclusion of samples with immoderately low bacterial biomass. Following completion of microbiome sequence preprocessing and decontamination, our final cohort consisted of 137 women, 73 of whom donated either T tissue, AN tissue, or both, 49 women who donated H tissue, and 15 women who donated PD tissue (Fig. 1). Collectively, these 137 women donated 159 breast tissue samples (H = 49, PD = 15, AN = 49, and T = 46) to be analyzed for microbiota composition and function.

### DNA extraction.

We extracted DNA from all 165 mammary tissue samples using the Qiagen AllPrep PowerFecal DNA/RNA kit (Qiagen, Hilden, Germany) by following the manufacturer’s guidelines, with the following modifications to the lysis procedure. In a sterilized tissue culture hood, samples were cut into 50-mg pieces and placed into labeled bead tubes containing approximately 650 μL of lysis buffer, 50 μL of proteinase K, and 25 μL of dithiothreitol (DTT). The samples were then placed in an ~70°C water bath for approximately 1 h with periodic vortexing until the entirety of the breast tissue was lysed. Following tissue lysis, the samples were homogenized twice in 2-min intervals using a bead mill. Following bead beating, DNA was isolated using the manufacturer’s instructions. DNA was stored at –80°C until library preparation and sequencing.

In addition to the DNA isolation from the mammary tissues, we also isolated DNA from a positive control (ZymoBIOMICS microbial community standard [catalog no. D6300]) and performed 9 extractions from negative controls (see Fig. S6A to C in the supplemental material). Approximately two extraction negative controls were isolated for each DNA extraction kit utilized in this study. The extraction negative controls consisted solely of the reagents and buffers used for DNA isolation to account for any possible reagent contamination. These negative controls were subjected to all steps of the sample preparation process (extraction, library preparation, and sequencing). These negative extraction controls, along with the positive-control microbial standard, represent published strategies to account for possible reagent contamination in low-biomass samples (25, 26).

10.1128/msystems.01489-21.6 FIG S6 Positive controls, decontamination, and pruning of the ASV table. DNA was isolated from a positive microbial standard (ZymoBIOMICS microbial community standard [catalog no. D6300]) using the protocol outlined in the text. The phyla and family present within our extracted control (A and B) reflect the composition of the ZymoBIOMICS microbial standard at the genus level (C). Following assessment of the positive control, any contaminated sequences identified in the negative extraction controls (*n* = 9) were removed from all samples using the decontam package in R. Removal was based on prevalence of the ASVs in samples relative to the prevalence of the ASVs in negative controls. This process removed 775,278 reads from our analysis. (D) Total read counts before and after decontamination. (E and F) Read counts of H, PD, AN, T, positive control (pos), and blanks before (E) and after (F) decontamination. (G) Phylum distribution in H, PD, AN, and T tissues after pruning ASVs with fewer than 20 reads and removal of samples with fewer than 10,000 reads. Download FIG S6, PDF file, 0.3 MB. Copyright © 2022 Hoskinson et al. 2022 Hoskinson et al. https://creativecommons.org/licenses/by/4.0/ This content is distributed under the terms of the Creative Commons Attribution 4.0 International license.

### 16S microbial community analysis. (i) Library preparation and sequencing.

Extracted DNA from 165 tissue samples, 9 negative extraction controls, and 1 positive control was submitted to the University of California Davis Host Microbe Systems Core Lab for library preparation and sequencing. Samples were subjected to a nested library preparation procedure. First, primers spanning the full-length 16S rRNA gene (27F/1492R) are applied to amplify and enrich for the full-length bacterial 16S rRNA gene. Second, a traditional two-step PCR process is applied to these full-length 16S amplicons to amplify the V3-V4 regions (primers 319F and 806R) of the 16S gene and add indices for sample identification after sequencing is complete. Amplicons resulting from this nested library preparation were quantified and subsequently pooled to equalize concentrations for Illumina MiSeq sequencing. In addition to the extraction negative controls, which were subjected to all sequencing preparation steps (extraction, library preparation, and sequencing), PCR-specific no template controls were included in the 16S enrichment and index PCR steps. These PCR-specific no template controls yielded no amplification. Following library preparation, samples were pooled and sequenced via Illumina MiSeq bidirectional paired-end sequencing (2 × 300 bp; Illumina, San Diego, CA, USA).

### (ii) Sequence preprocessing.

Overlapping paired-end reads were processed into amplicon sequence variants (ASVs) with DADA2 (27). Unique ASVs were aligned to the SILVA reference database and assigned taxonomy using the assignTaxonomy function as outlined in the DADA2 tutorial (27, 28). The only modification to the default DADA2 pipeline was in the length of the forward and reverse read trimming. Forward reads were trimmed to 260 bp, and reverse reads were trimmed to 190 bp prior to merging reads into contigs.

### RNA extraction and whole transcriptome profiling.

Transcriptome profiles of 219 H and PD subjects (*n*_H_ = 204, *n*_PD_ = 15) were analyzed for differential gene expression in two separate sequencing batches (available from Gene Expression Omnibus under GSE164641 [batch I] and GSE166044 [batch II]). Total RNA was isolated from fresh-frozen breast tissue biopsy specimens (150 to 200 mg) using the AllPrep DNA/RNA/miRNA kit (Qiagen). Tissues were homogenized by using 2-mL prefilled tubes containing 3-mm zirconium beads (number D1032-30; Benchmark Scientific), 350 μL lysis buffer, 2-mercaptoethanol, and BeadBug 6 homogenizer (Benchmark Scientific) in a cold room under the following conditions: 4,000 rpm for 45 s repeated twice with 90-s rest time. The concentration and quality of total RNA samples was first assessed using an Agilent 2100 Bioanalyzer. A RIN (RNA integrity number) of six or higher was required to pass the quality control.

Samples were then submitted to the Center for Genomics and Bioinformatics at IU Bloomington, where a cDNA library was prepared using the TruSeq stranded total RNA kit (Illumina) and sequenced using Illumina HiSeq4000. Reads were adapter trimmed and quality filtered using Trimmomatic ver. 0.38 (http://www.usadellab.org/cms/?page=trimmomatic), setting the cutoff threshold for average base quality score at 20 over a window of 3 bases. Reads shorter than 20 bases posttrimming were excluded. About 94% of the reads have both mates passing the quality filters. Using STAR version STAR_2.5.2b, 99% of cleaned reads were aligned to the human genome reference sequence GRCh38.p12 with gencode v.28 annotation (29).

### Statistical analysis.

All R code can be found in File S1.

10.1128/msystems.01489-21.7 FILE S1 R code. Download FILE S1, DOCX file, 0.03 MB. Copyright © 2022 Hoskinson et al. 2022 Hoskinson et al. https://creativecommons.org/licenses/by/4.0/ This content is distributed under the terms of the Creative Commons Attribution 4.0 International license.

### Metadata analysis.

Associations between clinical variables and cancer status were analyzed via logistic regression using the *glm* package in R (*n* cancer = 73, *n* healthy = 49, *n* prediagnostic = 15, *n* total = 137) (Table 1) (30). The *P* value cutoff for significance was 0.05.

### Microbial composition analysis.

Raw ASVs and taxa used in the following analyses are available at https://datadryad.org/stash/share/qq_3ZPf-f_QVPyhIEtbiy8AJm2_rYwHhyY9cno87YLY. Any contaminated sequences identified in the negative extraction controls (*n* = 9) were removed from all samples using the *decontam* package (31). This removal was based on the prevalence of the ASV in the samples relative to the prevalence of the ASV in negative controls. This process removed 775,278 reads from our analysis. We also removed all nonbacterial reads (Fig. S6D to F). After decontamination, samples with fewer than 10,000 reads were removed from the data set, and the data were rarefied to the smallest sample depth of 18,441 reads. After rarefaction, we constructed a phylogenetic tree using the APE package in R (32). Next, we used the *Phyloseq* and *vegan* packages (33, 34) in R to analyze alpha diversity based on the Chao1 and Shannon diversity indices and beta diversity based on the weighted and unweighted UniFrac distances. After analysis of alpha and beta diversity, ASVs with fewer than 20 reads were removed from this data set. In total, 8,225,367 reads passed decontamination and pruning (Fig. S6G). ASVs were transformed to percent abundance per sample prior to construction of relative abundance plots in *Phyloseq* and analysis with MaAsLin2 and LEfSe (35, 36). Phylum abundance plots were constructed using all ASVs. We isolated the top 100 ASVs prior to constructing relative abundance plots at the family level. For identification of differentially abundant taxa in PD, AN, and T tissue relative to H tissue, we used a consensus-based approach, employing both MaAsLin2 and LEfSe (35, 36). MaAsLin2 is a multivariate statistical model used to identify associations between microbial taxa or functional features and clinical metadata (30). We used MaAsLin2 under default settings with a *q* value threshold of 0.25 (Benjamini-Hochberg adjustment) for identification of differentially abundant ASVs. LEfSe uses the nonparametric Kruskal-Wallis test to identify significantly differentially abundant taxa between groups and then employs linear discriminant analysis (LDA) to estimate effect sizes of each identified microbial feature (36). LEfSe analysis was conducted on the Galaxy server (https://huttenhower.sph.harvard.edu/galaxy/) using the following settings: alpha ≤ 0.05, 2 for LDA threshold, and one-against-all strategy for multiclass analysis. The betadisper test in *vegan* was used to determine relative heterogeneity of the H, PD, AN, and T microbiota (34). The betadisper function analyzes the variance of each sample from the centroid and returns statistically significant findings (*P* ≤ 0.05) when comparing dispersal between tissues. The Shapiro-Wilk test for normality was applied to Chao1, observed ASV counts, and Shannon diversity indices, with a *P* value of ≤0.05 indicating that the samples are not normally distributed. Based on this test for normality, repeated-measures analysis of variance (ANOVA) and *post hoc* Tukey’s tests were applied to determine statistical differences in Shannon diversity indices between tissue types, and linear regression models were applied to analyze statistical differences in Chao1 diversity indices and observed ASV counts. All plots were created using the *ggplot2* package (37).

### Microbial functional analysis.

We used PICRUSt2 under default settings to infer a profile of putative microbial functions (via metagenome prediction) from the 16S rRNA after decontamination and removal of nonbacterial reads (14). Representative sequences were analyzed using PICRUSt2 and classified against the Kyoto Encyclopedia of Genes and Genomes (KEGG) Database according to 97% similarity (12, 13). This analysis provides insight into the molecular and metabolic function of microbiota in the breast tissue. We identified 7,170 KEGG orthologies (KO), otherwise referred to as nodes or steps in KEGG pathway maps, using this prediction software. Each was assigned a KO identifier indicating its status as a functional ortholog, done by inserting our KO results into the KEGG orthology data-oriented entry point of the KEGG orthology annotation ortholog table (OT). Within each KEGG database OT entry for the KO numbers, both the predicted general metabolic function and specific metabolic activity were assigned to the corresponding KOs. The same samples removed from the ASV table for microbiome compositional analysis (samples with less than 10,000 reads) were also removed from the PICRUSt2 data set before identification of differentially abundant microbial pathways. These data were relativized, and we used MaAsLin2 (same settings as those described above) and LEfSe (same settings as those described above) to identify KOs associated with the breast tissue type (PD, AN, and T) relative to H tissue (35, 36).

### Correlation analyses between host genes and microbial taxa and genes.

RNA from 219 H and PD subjects (*n*_H_ = 204, *n*_PD_ = 15) was analyzed for differential gene expression in two separate sequencing batches (available from Gene Expression Omnibus GSE164641 [batch I] and GSE166044 [batch II]) using *Deseq2*. To control for batch effect, we included a categorical variable for batch when conducting the differential expression analysis in *Deseq2* (38). Only genes identified as differentially expressed (DE) when controlling for the batch were included in our correlation analyses with the microbiome data. We identified 48 DE genes (alpha < 0.1) between H and PD subjects. Next, we isolated ASVs identified via either MaAsLin2 (*q *≤ 0.25) or LEfSe (*P* ≤ 0.05) as differentially abundant in PD and H tissue from subjects for whom we also had microbiome data from the decontaminated and pruned ASV table. Using the *psych* package in R, we conducted a Spearman’s rank correlation analysis between DE host genes and these bacterial ASVs for the PD and H groups separately (39). We then isolated the KOs identified as differentially abundant from the PICRUSt2 predicted functional metagenome between the PD and H tissues via either MaAsLin2 (*q *≤ 0.25) or LEfSe (*P* ≤ 0.05). Similar to the gene-ASV analysis, we used the *psych* package in R to conduct a Spearman’s rank correlation analysis between DE host genes and bacterial KOs for the PD and H subjects (*n*_H_ = 6, *n*_PD_ = 6) from whom we also had microbiome data separately (39). All correlations reported are statistically significant at an adjusted *P* value cutoff of 0.05 (Holm’s method). Data used in these correlation analyses can be found in Table S2.

A STORMS checklist is available at https://datadryad.org/stash/share/qq_3ZPf-f_QVPyhIEtbiy8AJm2_rYwHhyY9cno87YLY (40).

### Ethics approval and consent to participate.

Collection of breast tissue was performed by the KTB and IUSCCC Tumor Bank under a protocol approved by the Indiana University Institutional Review Board (IRB protocol number 1011003097 and protocol number 11438, respectively). All analyses performed on these tissues were approved by Pepperdine University’s Institutional Review Board (protocol number 18-07-837).

### Data availability.

All fastq files for 16S sequencing were deposited in the NCBI Sequence Read Archive (SRA) (accession number PRJNA723425). Transcriptome data were deposited in Gene Expression Omnibus (GEO) under GEO numbers GSE164641 (batch I) and GSE166044 (batch II). All R code used to analyze these data sets can be found in File S1. Relevant data sets are available as additional files (MaAsLin2 findings and correlation data) or by accessing the following link: https://datadryad.org/stash/share/qq_3ZPf-f_QVPyhIEtbiy8AJm2_rYwHhyY9cno87YLY (link to the Dryad repository, which includes metadata for Table 1, raw ASVs, and the raw PICRUSt2 data: https://doi.org/10.5061/dryad.9s4mw6mjd). We encourage requests of any other data sets or information that would be helpful to those aiming to validate these findings.
