# Supplementary figures and images for: Composition and Functional Potential of the Human Mammary Microbiota Prior to and Following Breast Tumor Diagnosis
Source: mSystems. 2022 Jun 1;7(3):e01489-21. doi: 10.1128/msystems.01489-21 (PMC9239270; doi:10.1128/msystems.01489-21)

A.

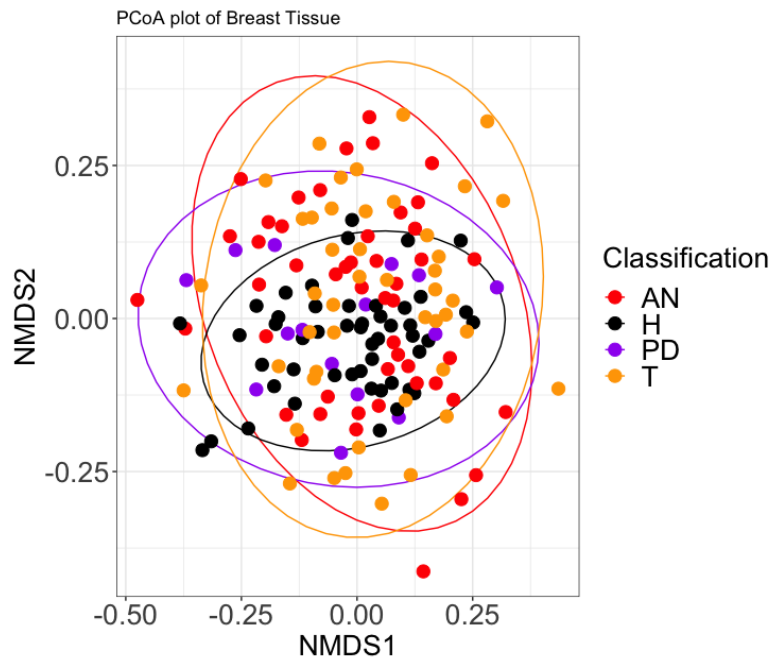

B.

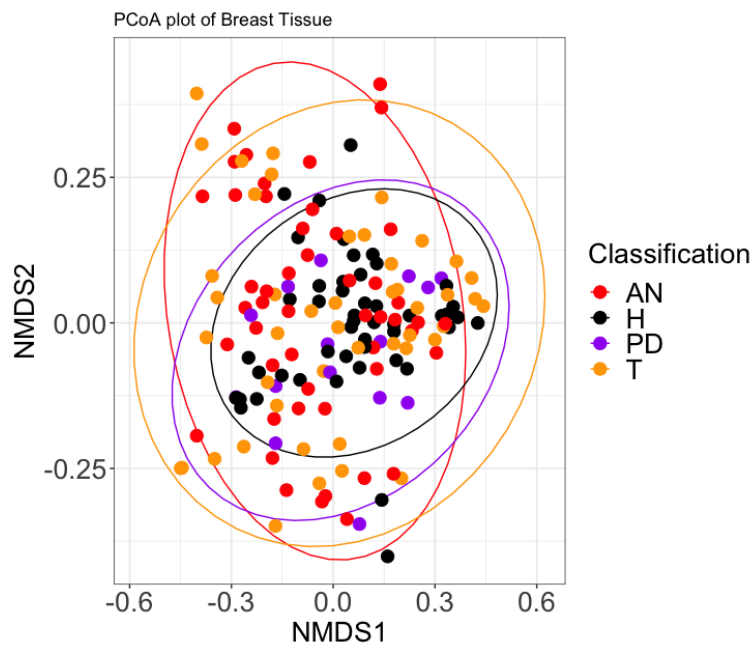

Supplement: FIG S1 [file msystems.01489-21-s0001.pdf]

A

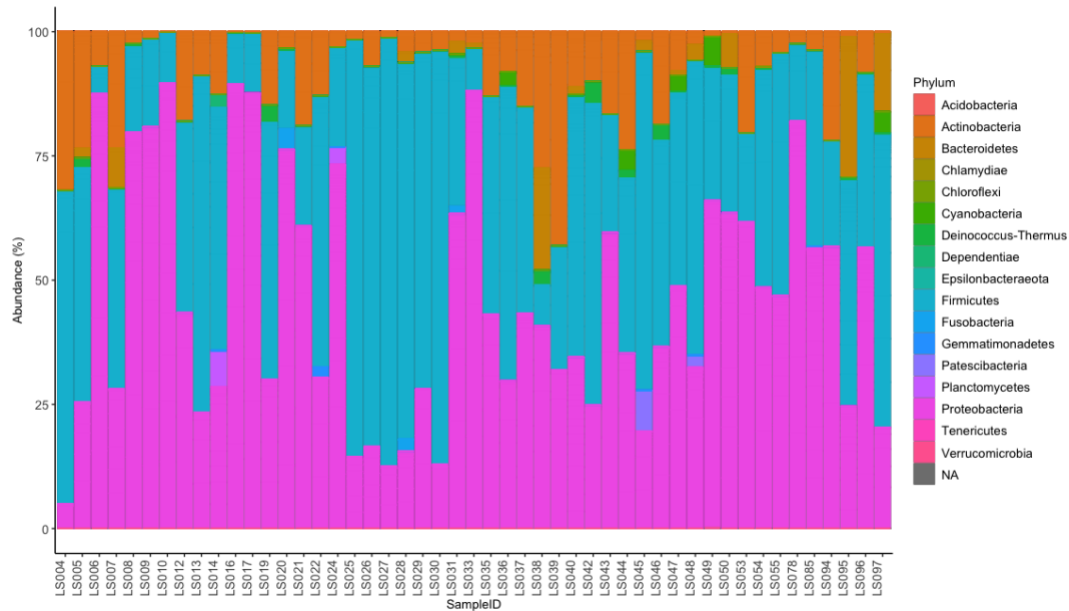

B

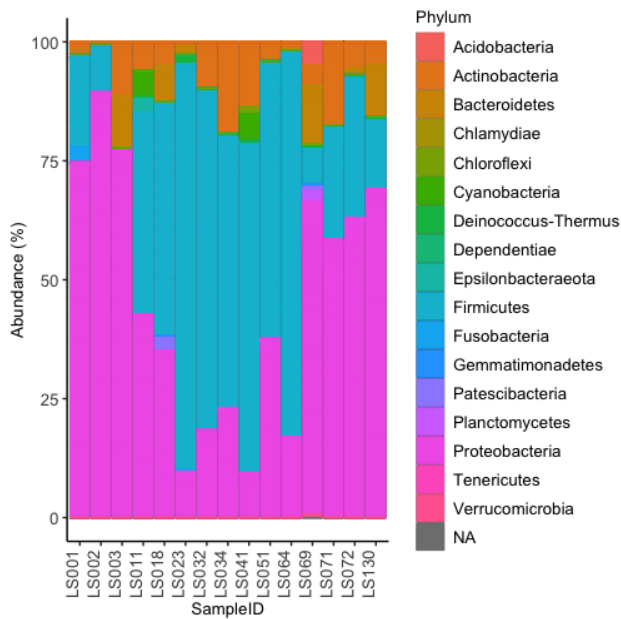

C

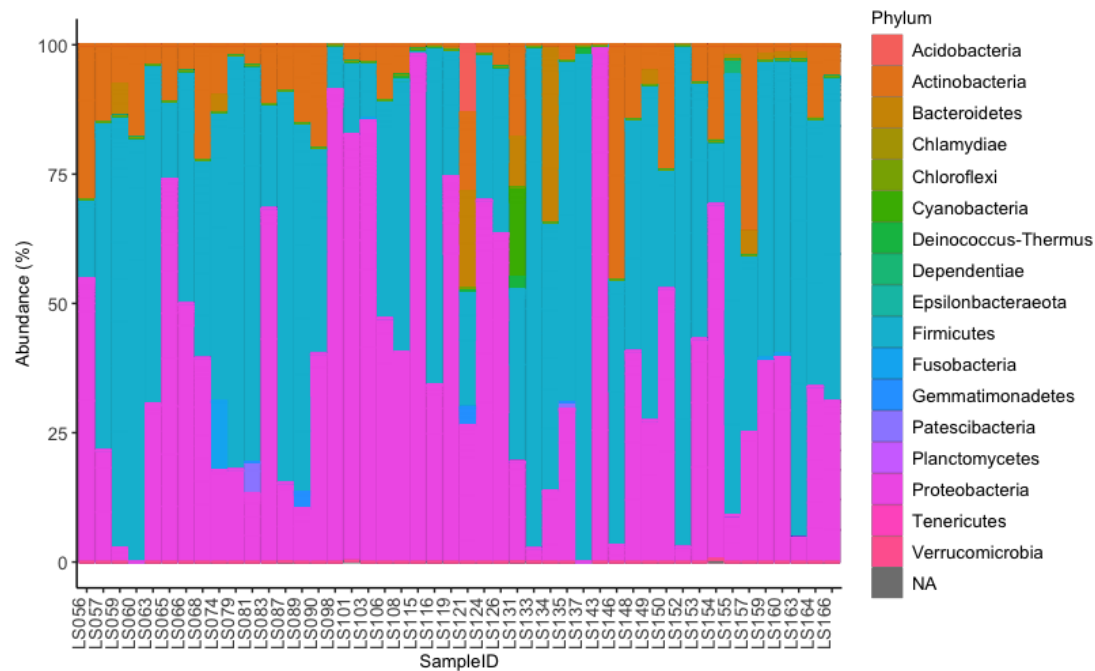

D

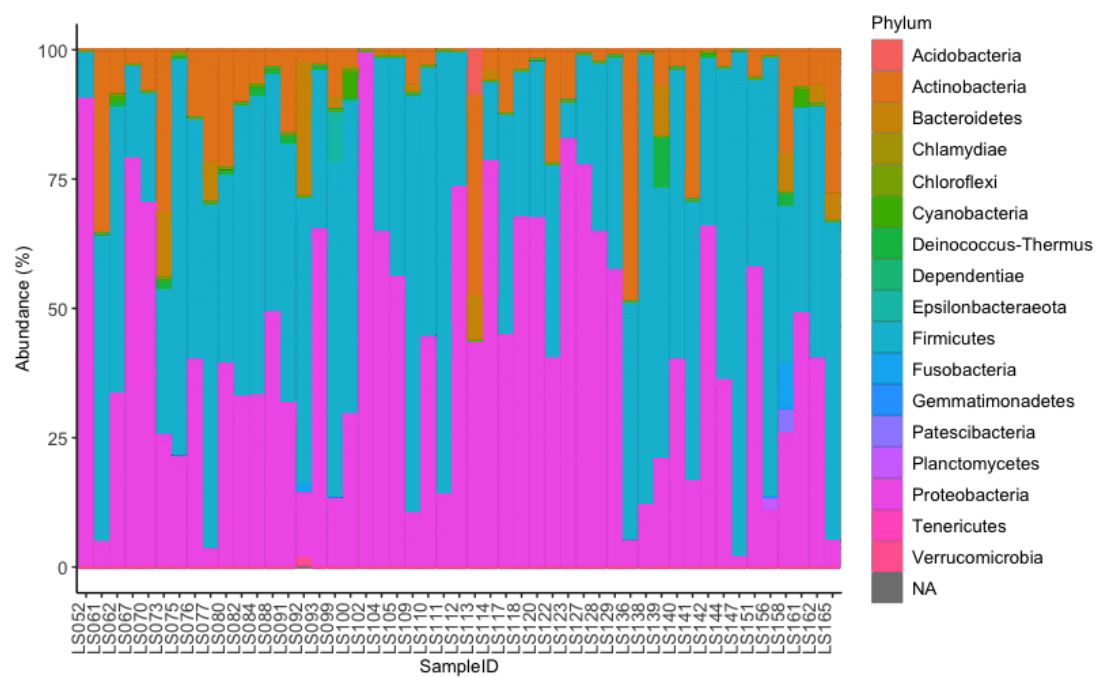

Supplement: FIG S2 [file msystems.01489-21-s0002.pdf]

A

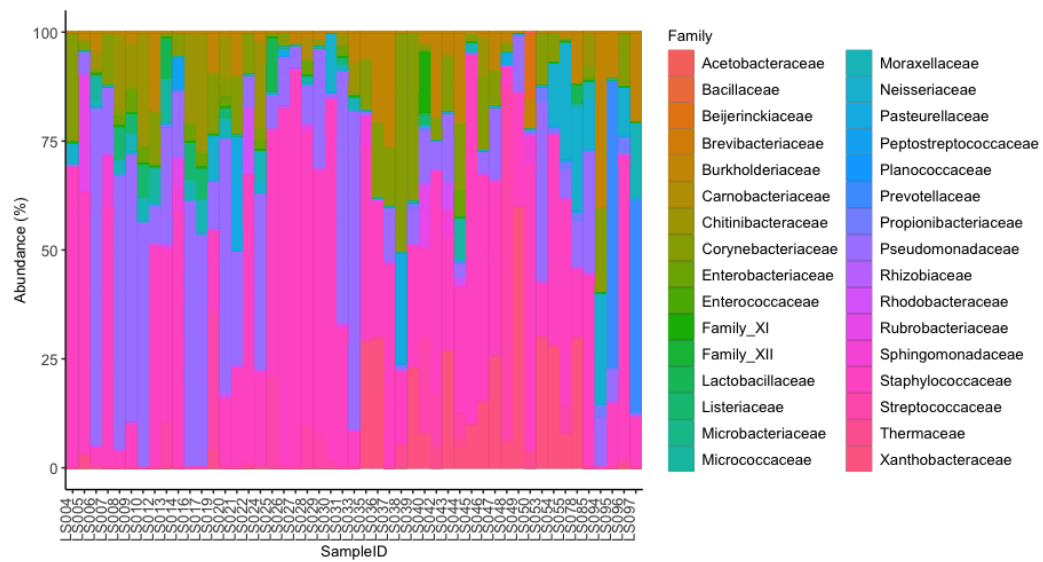

B

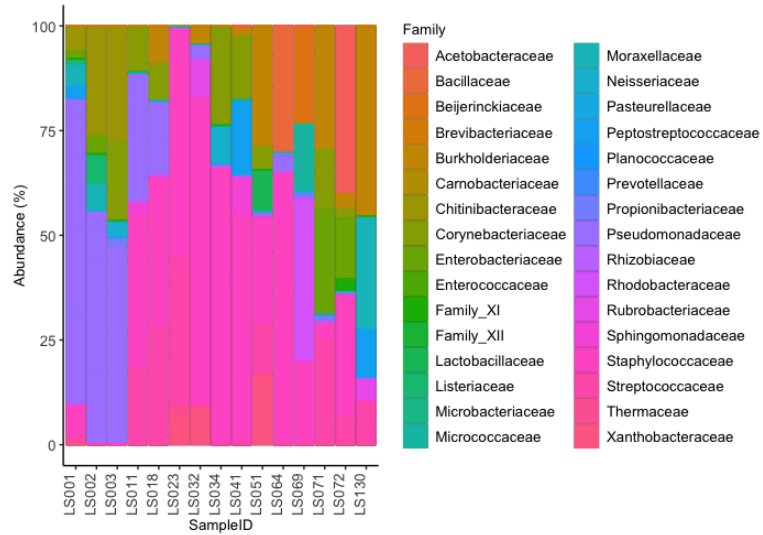

C

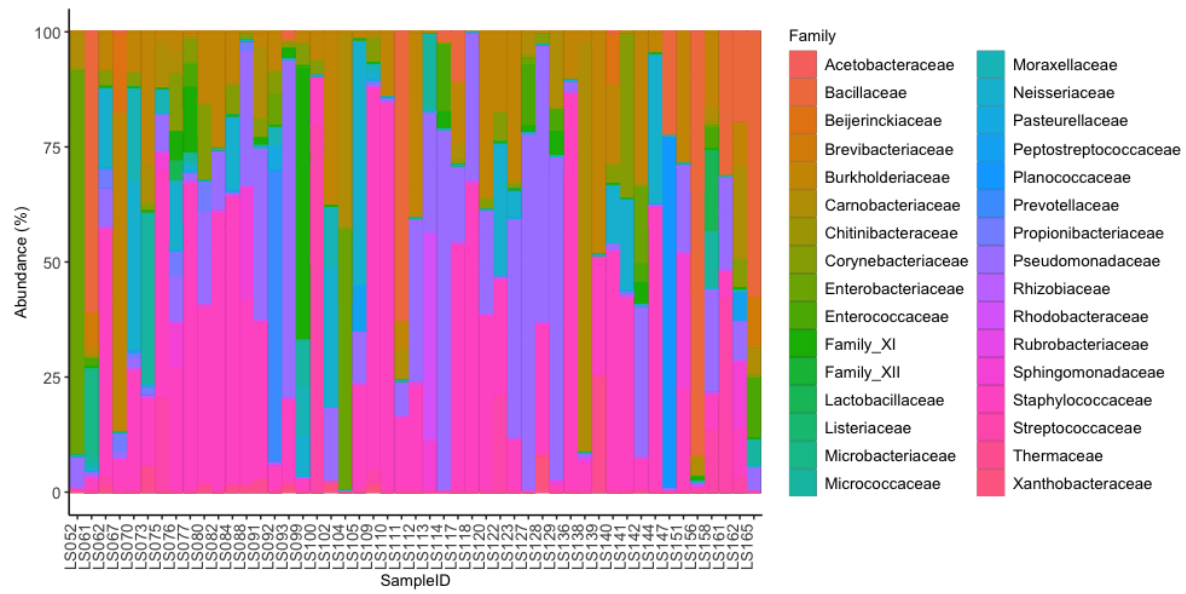

D

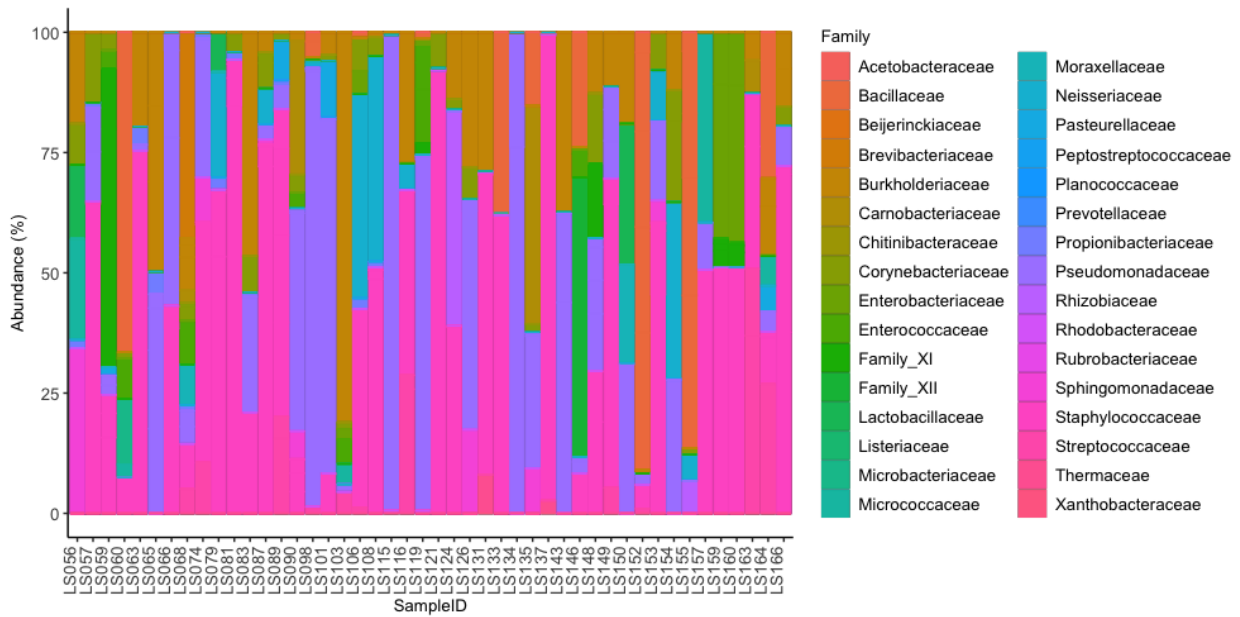

Supplement: FIG S3 [file msystems.01489-21-s0003.pdf]

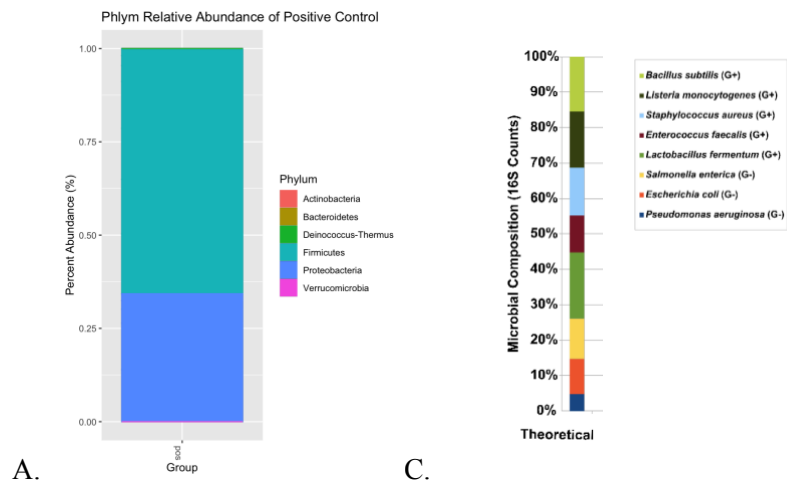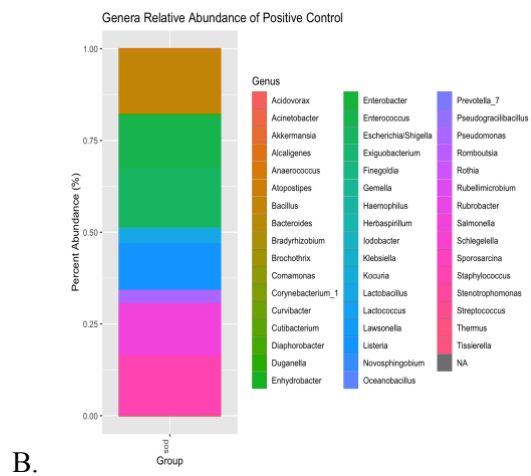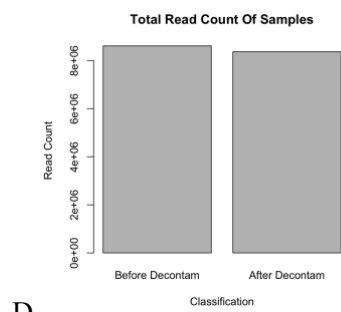

Read count of pre-decontaminated samples

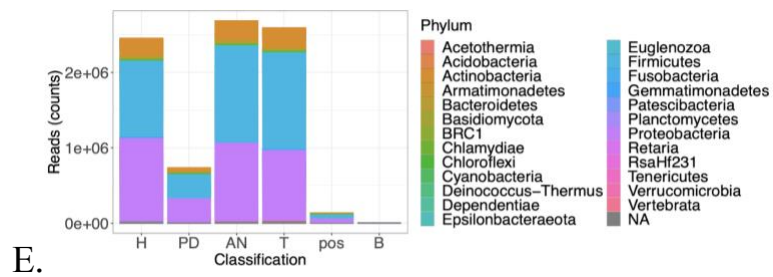

## Read count of decontaminated samples

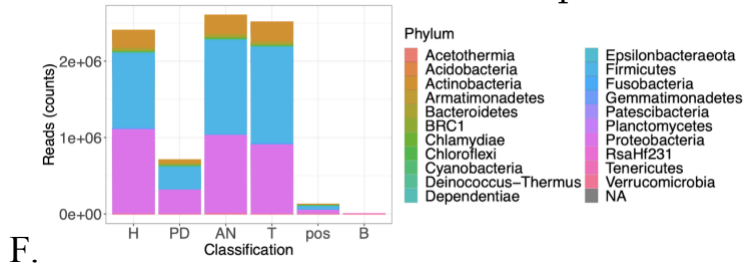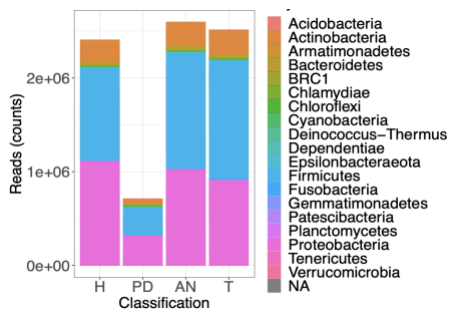

Supplement: FIG S6 [file msystems.01489-21-s0006.pdf]
