# Supplementary material for: Composition and Functional Potential of the Human Mammary Microbiota Prior to and Following Breast Tumor Diagnosis
Source: mSystems. 2022 Jun 1;7(3):e01489-21. doi: 10.1128/msystems.01489-21 (PMC9239270; doi:10.1128/msystems.01489-21)
Supplement: FIG S4 [file msystems.01489-21-s0004.pdf]

MaAsLin2-identified differentially abundant taxa

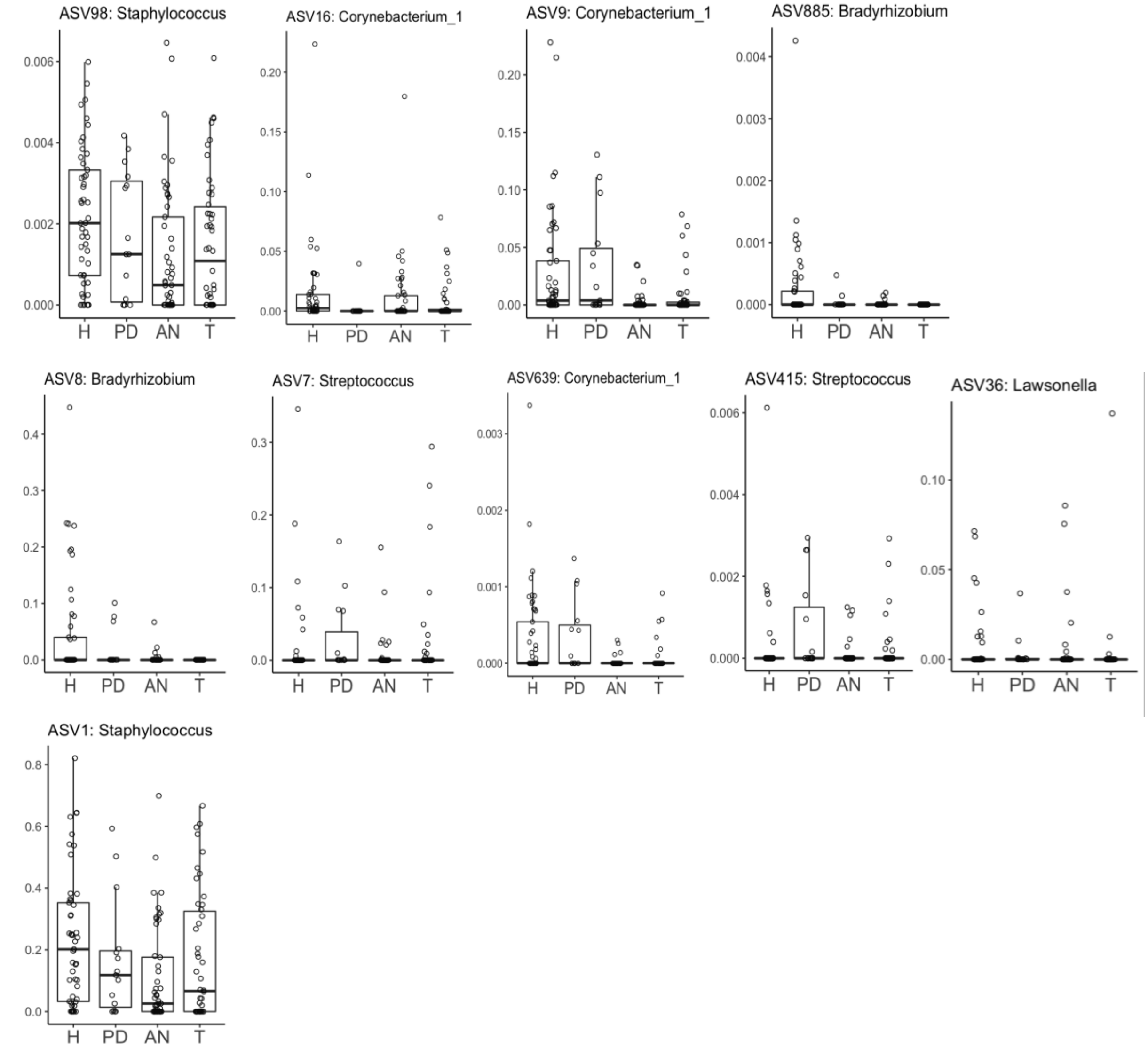

LefSE-identified differentially abundant taxa between H and PD

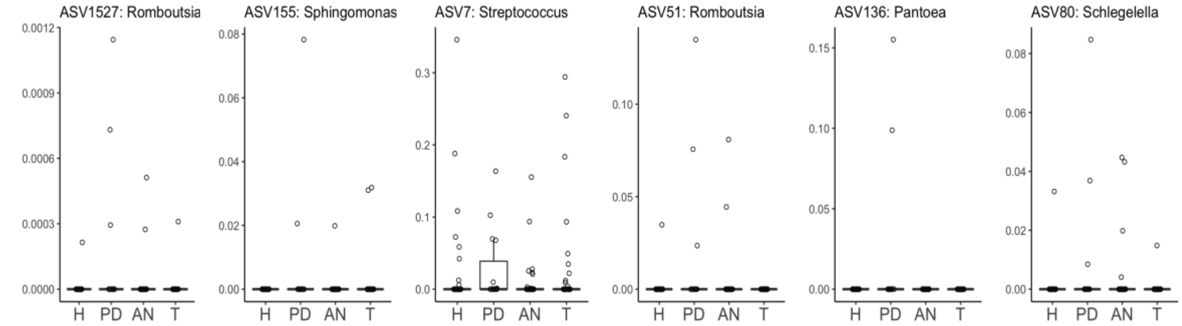

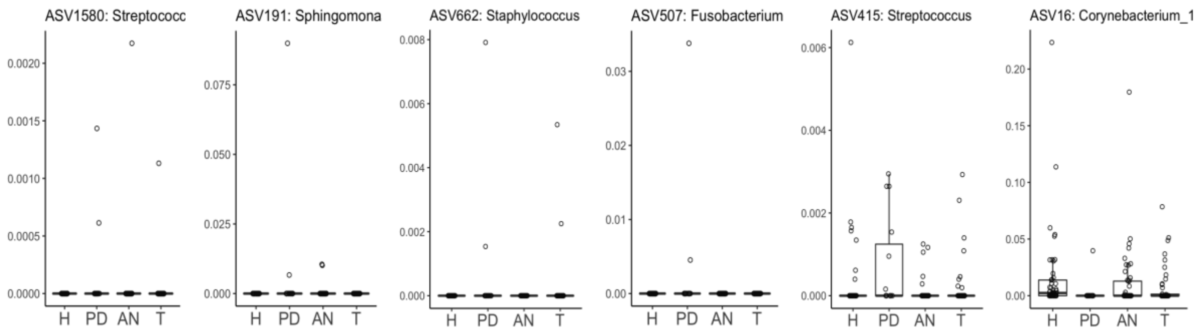

LefSE-identified differentially abundant taxa between H and AN (more abundant in AN)

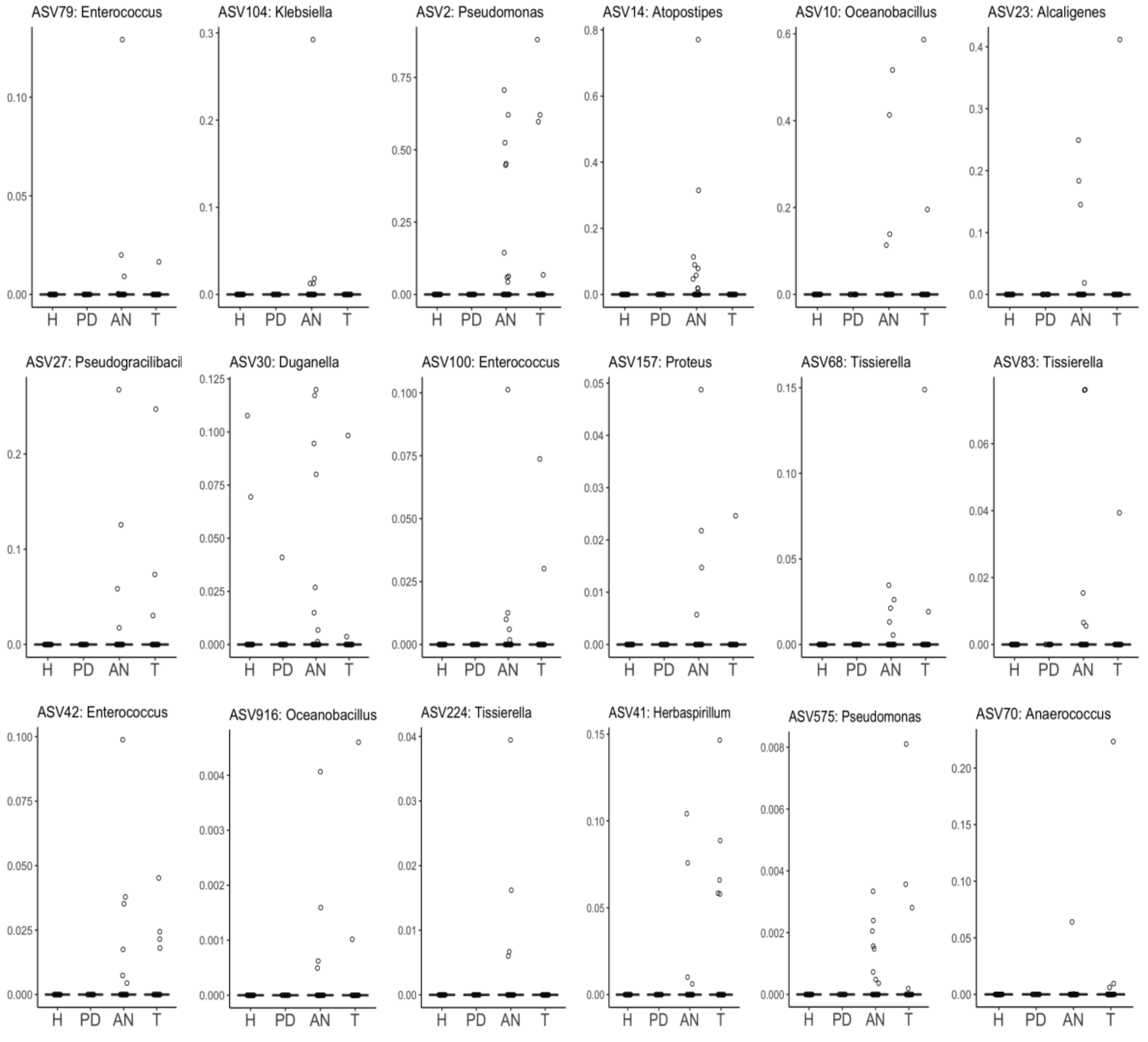

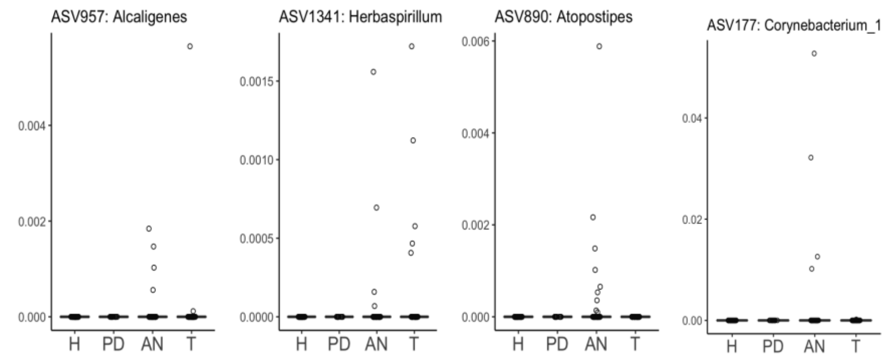

LefSE-identified differentially abundant taxa between H and AN (more abundant in H)

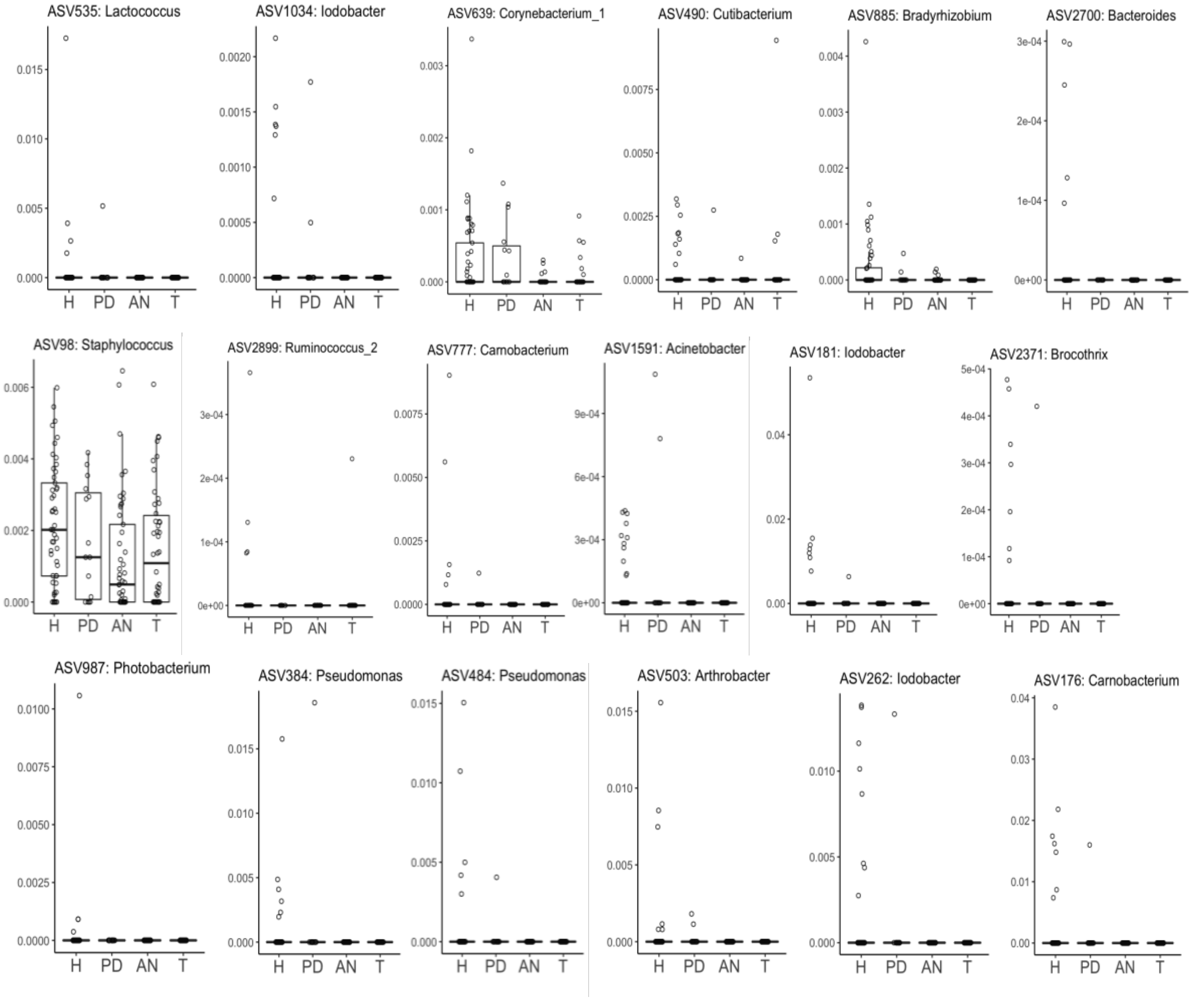

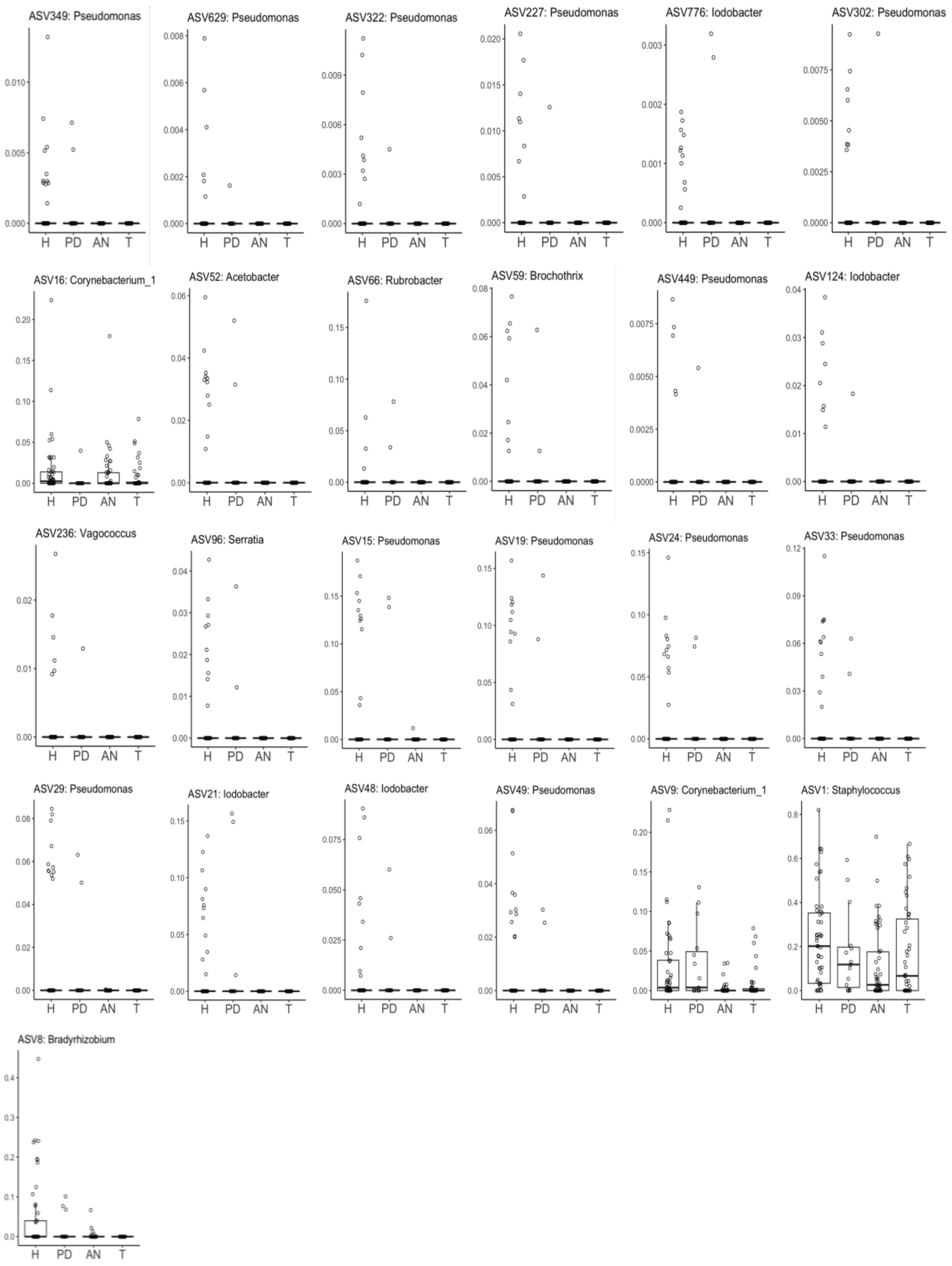

LefSE-identified differentially abundant taxa between H and T (more abundant in T)

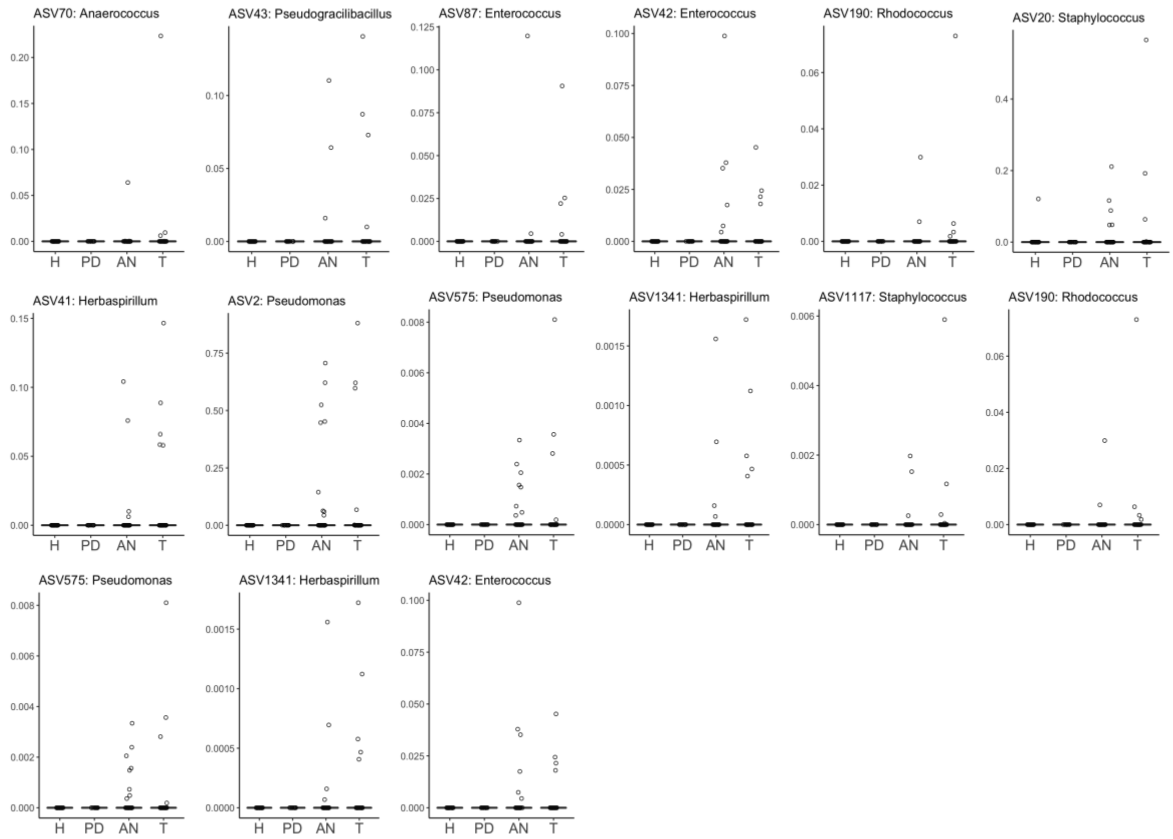

LefSE-identified differentially abundant taxa between H and T (more abundant in H)

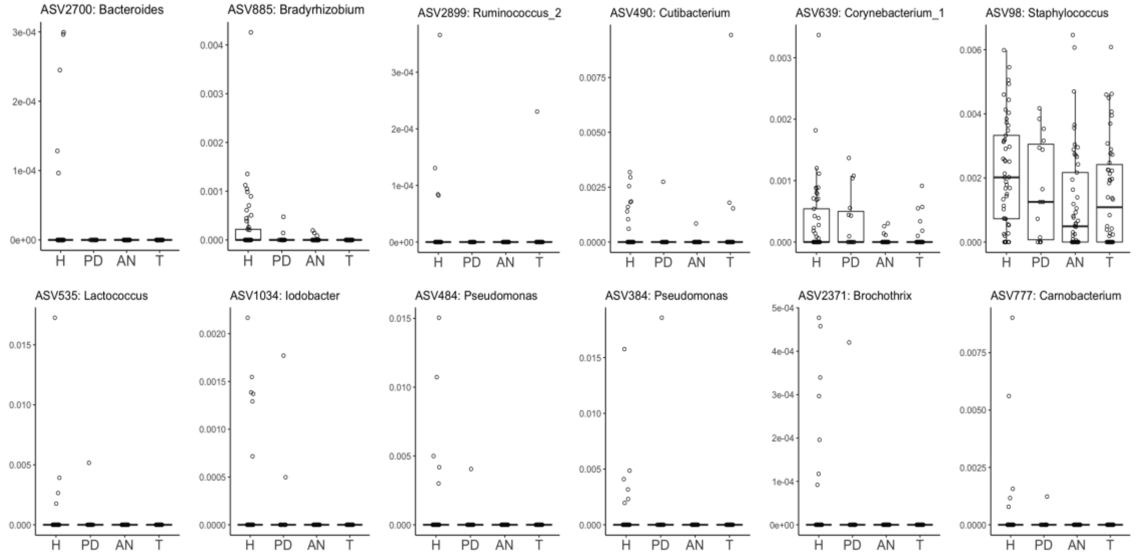

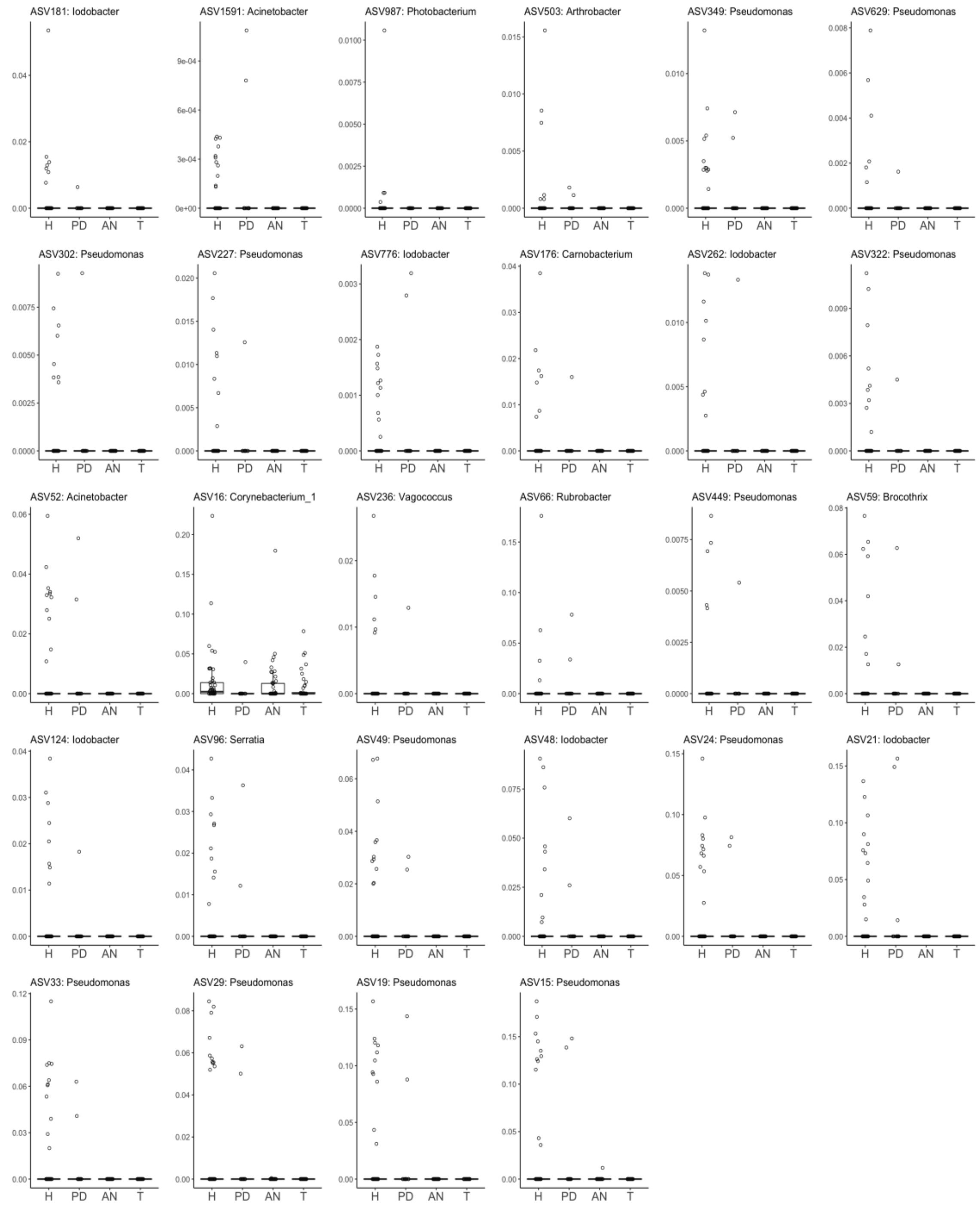

LefSE-identified differentially abundant taxa between H and T (more abundant in H) cont.

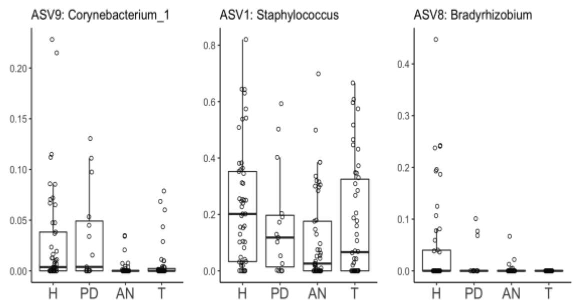

MaAsLin2-identified differentially abundant KEGGS

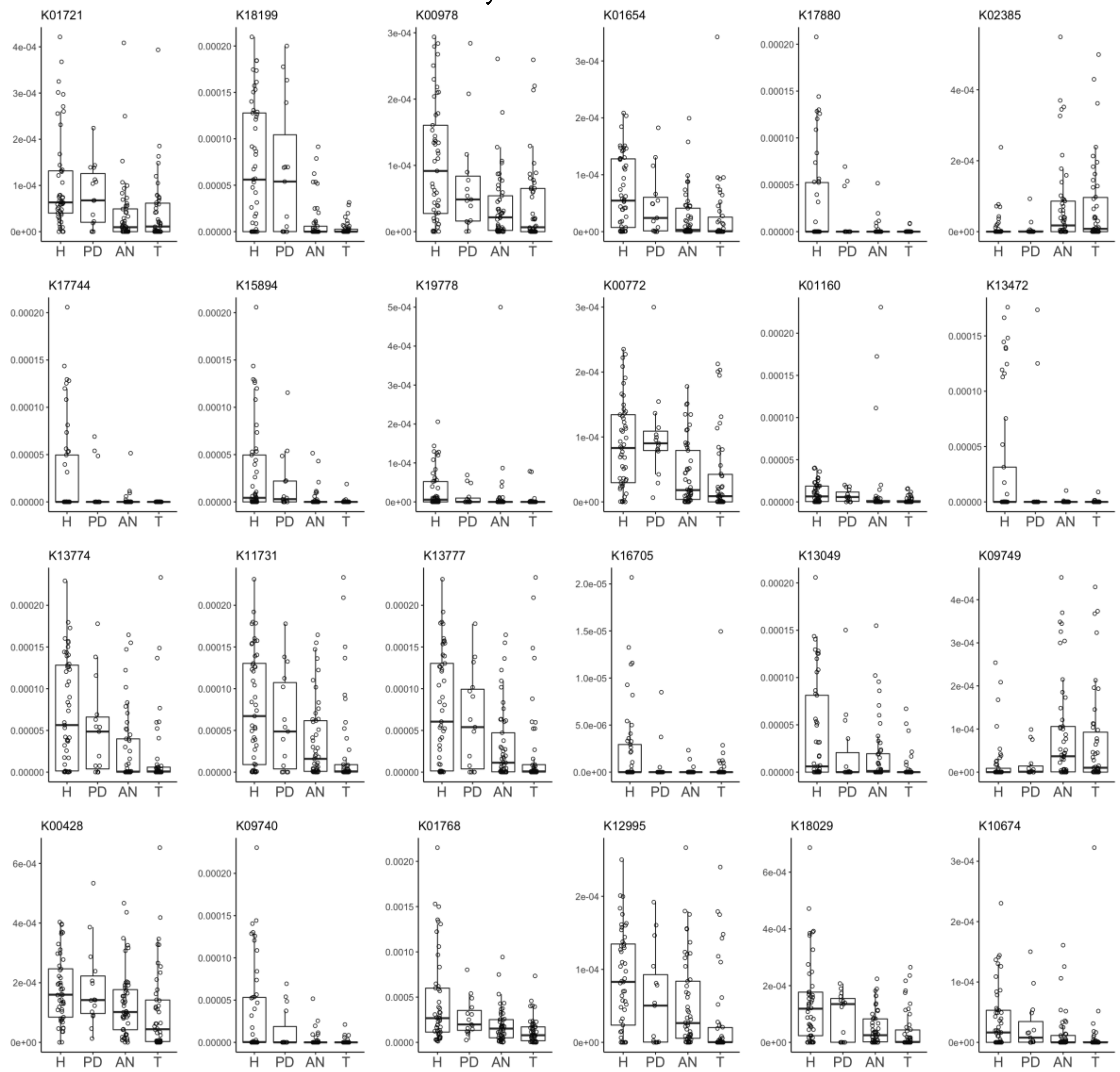

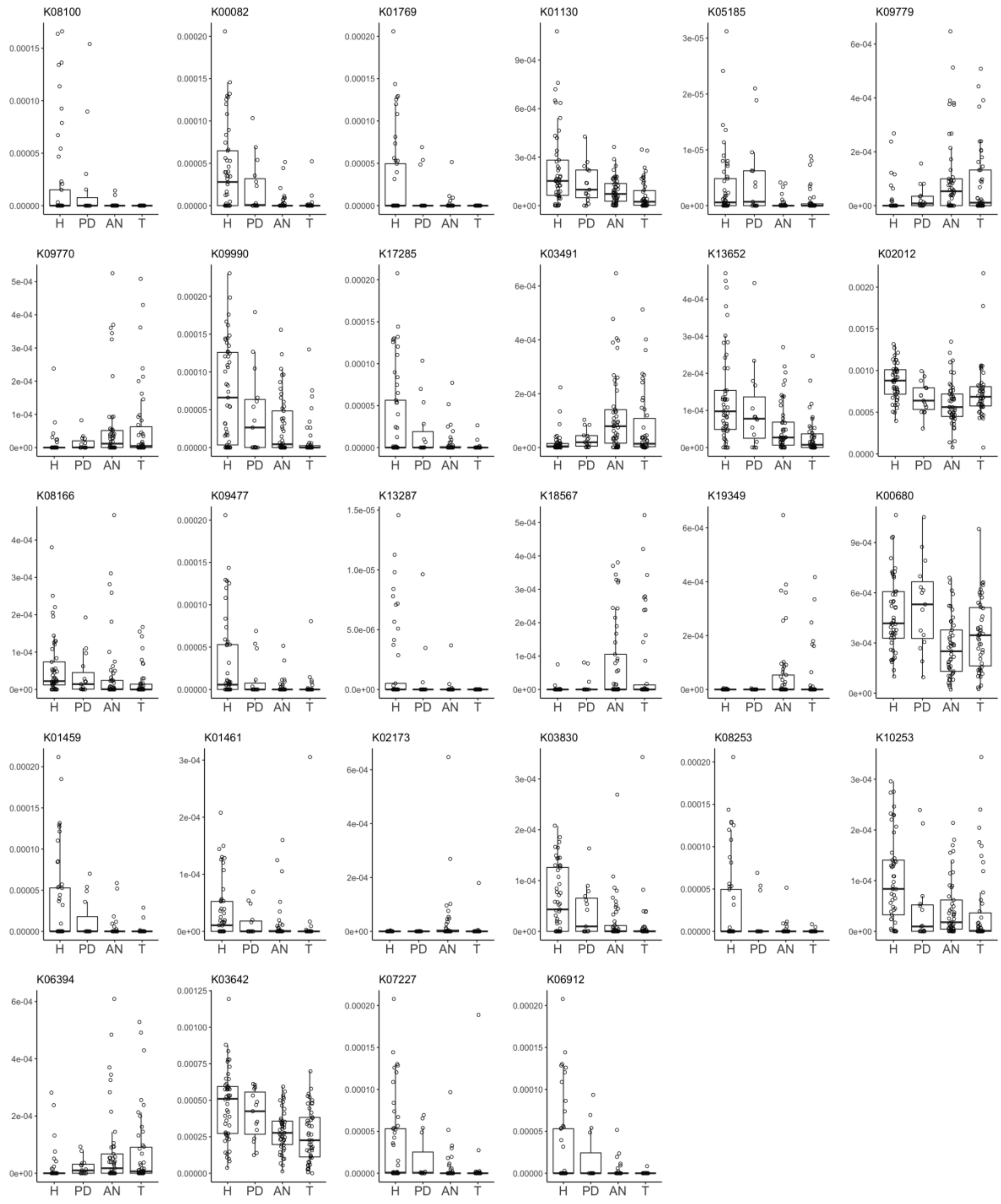

LefSE-identified differentially abundant KEGGs between H and PD (more abundant in H)

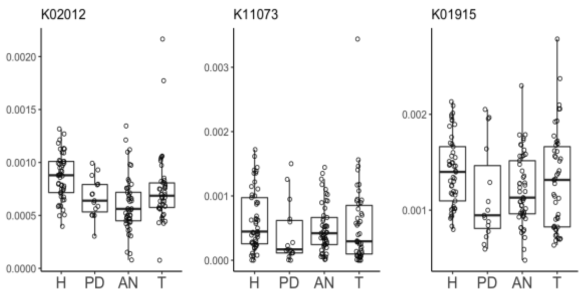

LefSE-identified differentially abundant KEGGs between H and AN (more abundant in AN)

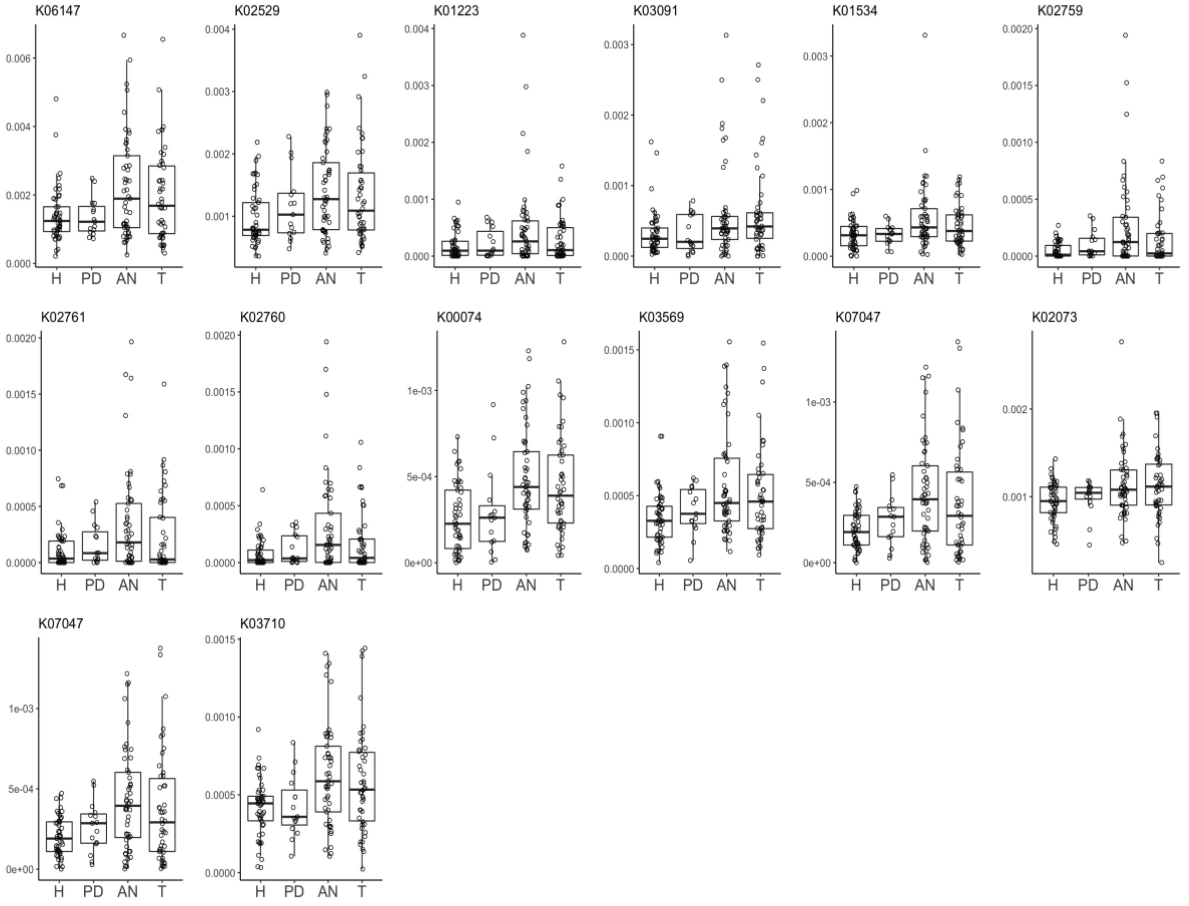

LefSE-identified differentially abundant KEGGs between H and AN (more abundant in H)

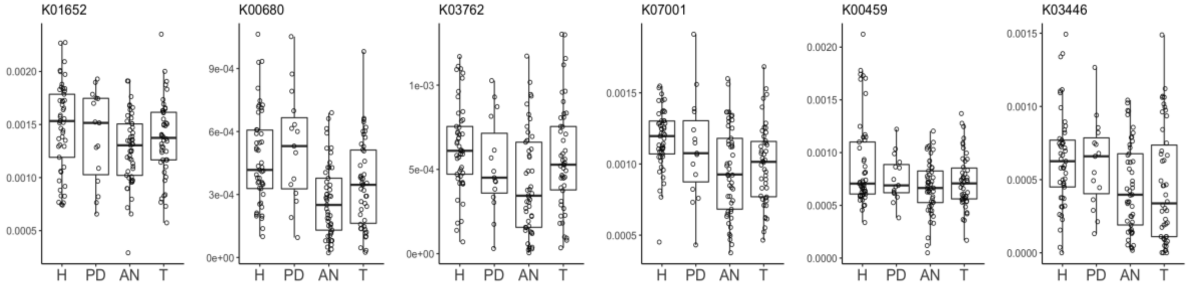

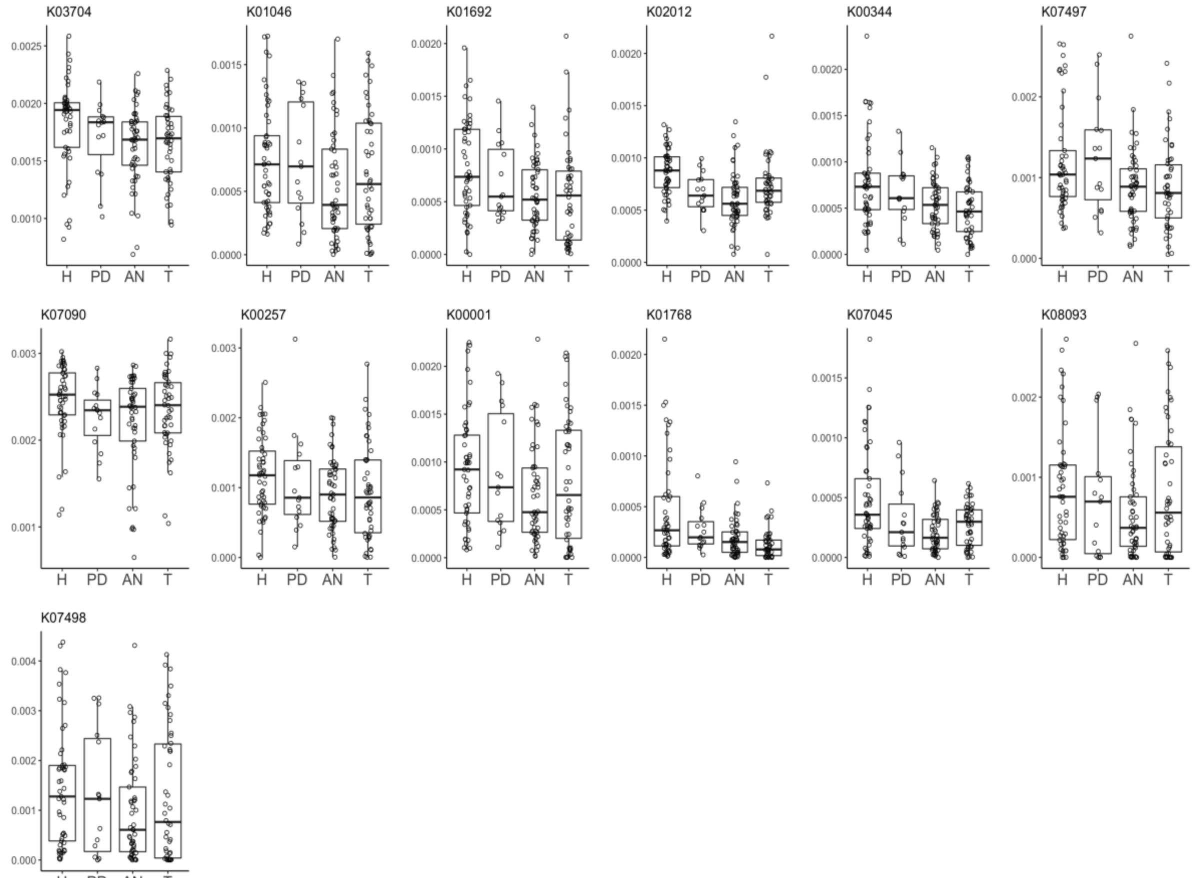

LefSE-identified differentially abundant taxa between H and T (more abundant in T)

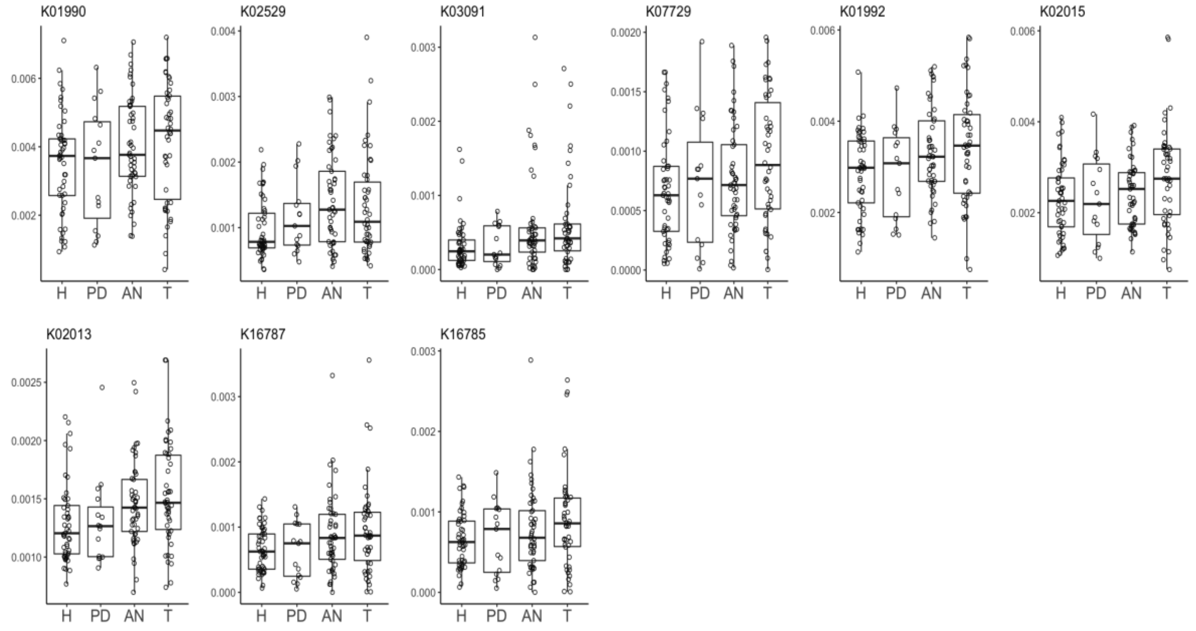

LefSE-identified differentially abundant taxa between H and T (more abundant

in H)

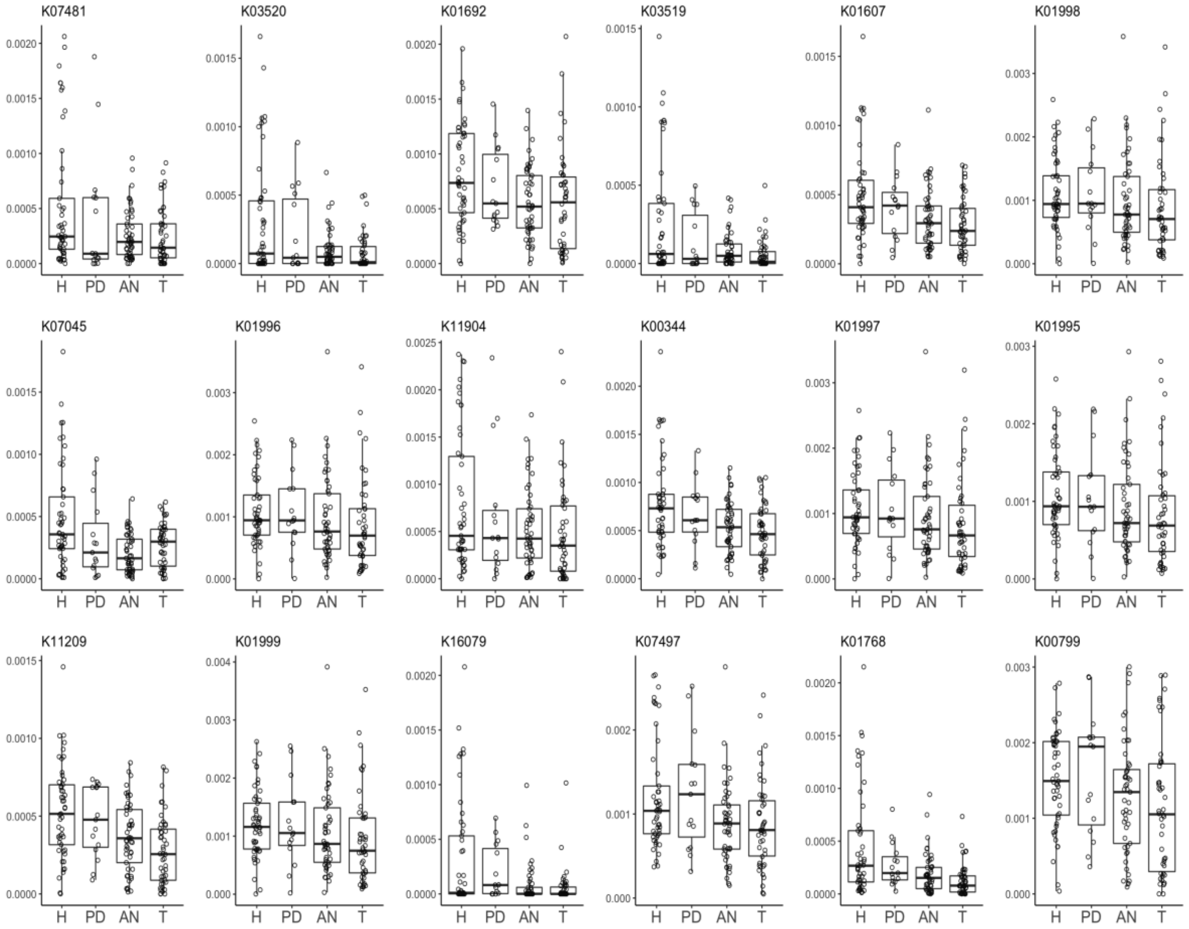
K07481
K03520
K01692
K03519
K01607
K01998
K07045
K01996
K11904
K00344
K01997
K01995
K11209
K01999
K16079
K07497
K01768
K00799
H
PD
AN
T
