# Supplementary material for: Composition and Functional Potential of the Human Mammary Microbiota Prior to and Following Breast Tumor Diagnosis
Source: mSystems. 2022 Jun 1;7(3):e01489-21. doi: 10.1128/msystems.01489-21 (PMC9239270; doi:10.1128/msystems.01489-21)
Supplement: FIG S5 [file msystems.01489-21-s0005.pdf]

KO-gene correlations in H group

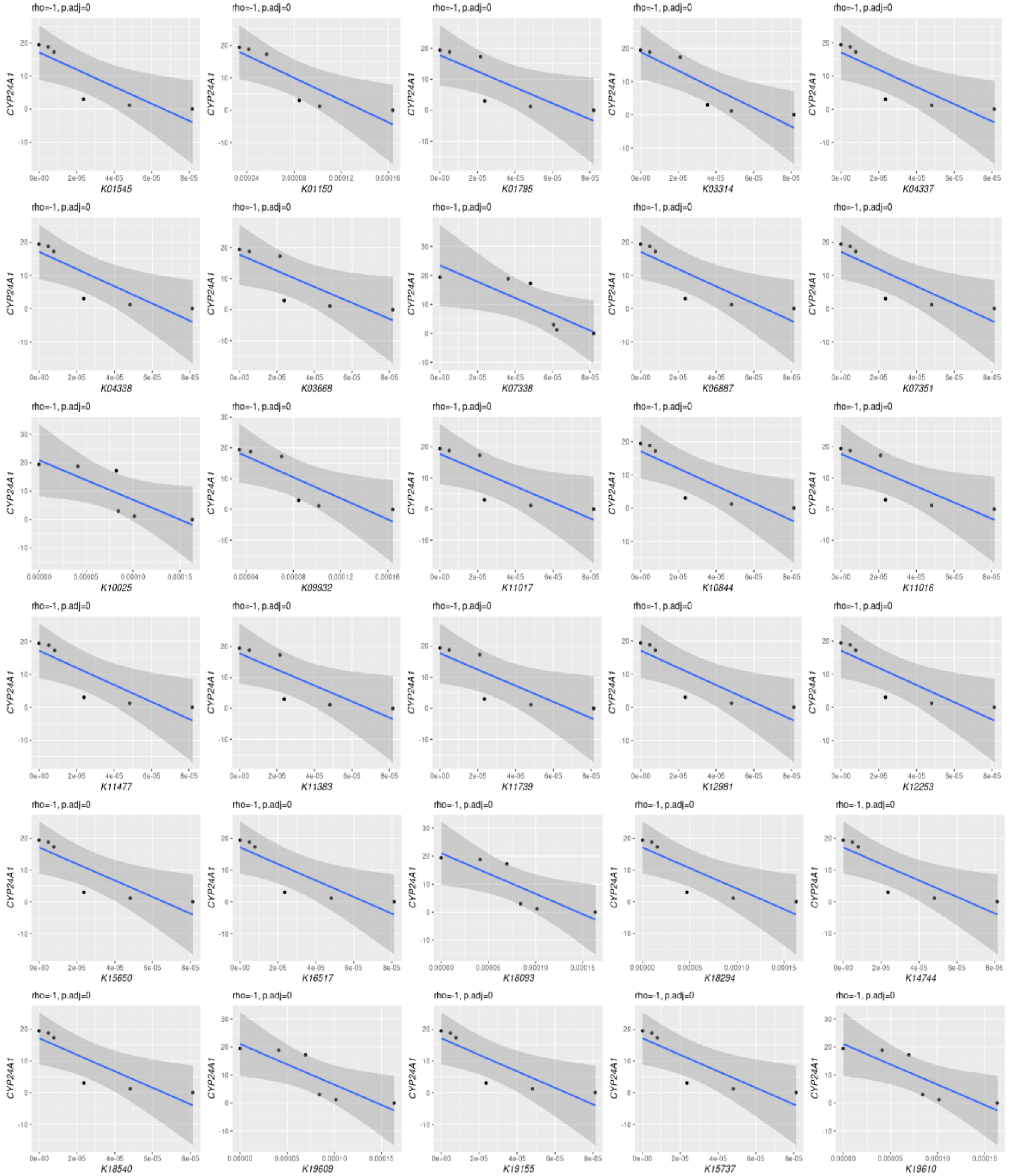

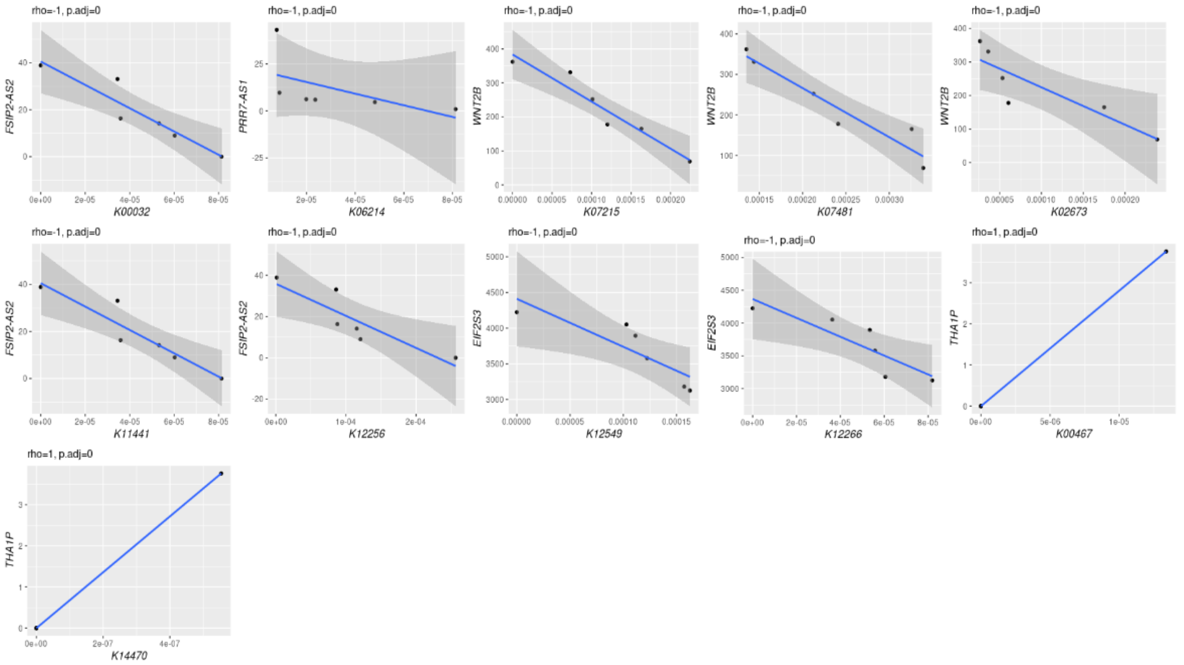

ASV-gene correlations in PD group

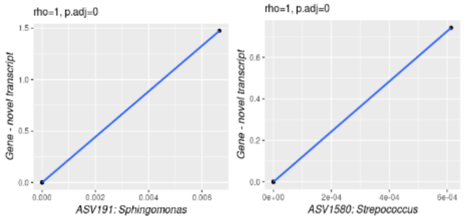

KO-gene correlations in PD group

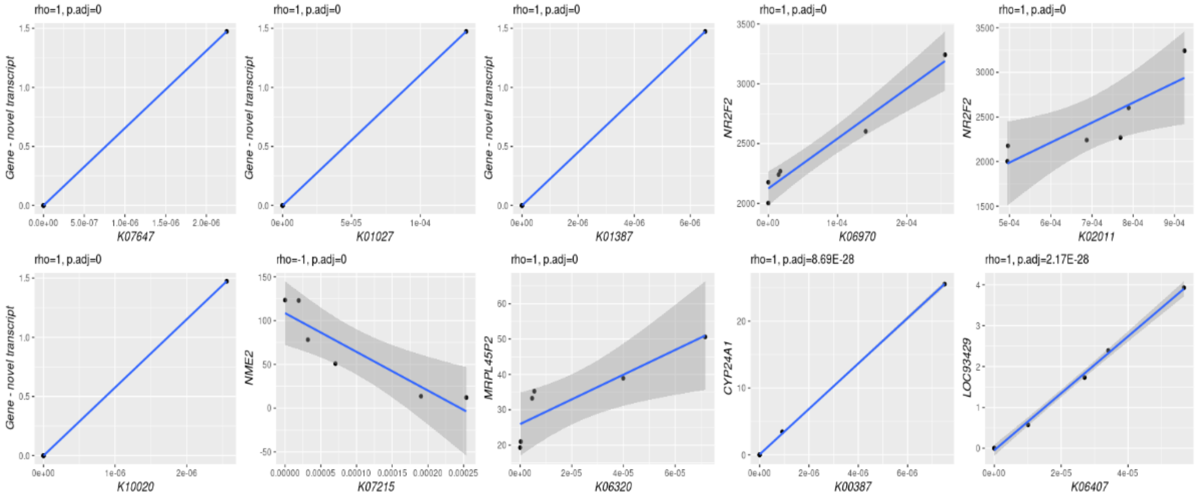

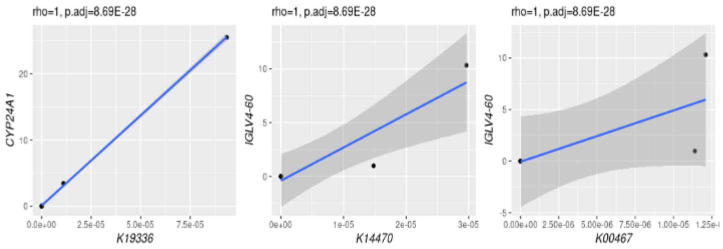
rho=1, p.adj=8.69E-28
CYP24A1
K19336
rho=1, p.adj=8.69E-28
IGLV4-60
K14470
rho=1, p.adj=8.69E-28
IGLV4-60
K00467
