# Supplementary material for: Composition and Functional Potential of the Human Mammary Microbiota Prior to and Following Breast Tumor Diagnosis
Source: mSystems. 2022 Jun 1;7(3):e01489-21. doi: 10.1128/msystems.01489-21 (PMC9239270; doi:10.1128/msystems.01489-21)
Supplement: FILE S1 [file msystems.01489-21-s0007.docx]

*#R Code for Hoskinson et al. 2021.*

library(phyloseq)

library(decontam)

library(ggplot2)

library(vegan)

library(nlme)

library(Maaslin2)

library(funrar)

*###################################################################################################*

*#DECONTAMINATION OF SAMPLE*

ps.ls <- readRDS("ps.Rds") #All data in Additional File 1

metadata <- read.csv("Metadata_AllGroups - Focus Data Used for Analyses.csv")

mapfile <- read.csv('mapfile_Stiemsma_2020.csv')

mapfile$GROUP[mapfile$GROUP == "C"] <- "T"

mapfile$GROUP[mapfile$GROUP == "N"] <- "AN"

mapfile <- subset(mapfile, GROUP != 'No Sample')

sample_data(ps.ls)$Classification <- as.factor(mapfile$GROUP)

sample_data(ps.ls)$Classification <- as.factor(sample_data(ps.ls)$Classification)

*#decontam*

tail(sample_data(ps.ls))

*#Designate blanks/negative controls ~ "B" refers to blank samples.*

sample_data(ps.ls)$is.neg <- sample_data(ps.ls)$Classification %in% "B"

*#Generate logical table of negative control samples in TRUE/FALSE format.*

contamdf.prev <- isContaminant(ps.ls, method="prevalence", neg="is.neg")

table(contamdf.prev$contaminant)

head(which(contamdf.prev$contaminant))

*#Threshold is 0.5 - will identify all sequences that are more prevalent in*

*#negative controls than in positive samples as contaminants.*

contamdf.prev <- isContaminant(ps.ls, method="prevalence", neg="is.neg", threshold=0.5)

table(contamdf.prev$contaminant)

*#Create new phyloseq object with newly decontaminated sequences.*

ps.noncontam <- prune_taxa(!contamdf.prev$contaminant, ps.ls)

ps.noncontam.onlybacteria <- subset_taxa(ps.noncontam, Kingdom == 'Bacteria')

*##############################################################################################*

*##Positive control - Figure S6*

pos <- subset_samples(ps.noncontam, SampleID%in% c("LS167"))

plot_bar(pos, "Classification", "Abundance", "Phylum")+

xlab("Classification") +

ylab("Reads (counts)") +

ggtitle("Read (counts) of decontaminated, pruned taxa and pruned samples") +

geom_bar(aes(color=Phylum, fill=Phylum),

stat="identity", position='stack') +

theme_bw() +

theme(axis.title.x=element_text(size=20)) +

theme(axis.title.y=element_text(size=20)) +

theme(axis.text.x=element_text(size=20)) +

theme(axis.text.y=element_text(size=20)) +

theme(legend.text=element_text(size=20)) +

theme(legend.title=element_text(size=20)) *#repeat for family*

*##Initial sum of reads prior to adjustments.*

sum(ps.ls@otu_table)

*#[1] 8610354*

sum(ps.noncontam.onlybacteria@otu_table)

*#[1] 8368201*

reads <- as.numeric(c("8610354","8368201"))

barplot(reads, main = "Total Read Count Of Samples",

xlab = "Classification",ylab = "Read Count",

names.arg = c("Before Decontam", "After Decontam"),

col = "grey",horiz = FALSE)

*#Figures S7. Visual comparison of contaminated and decontaminated data.*

ps.ls@sam_data$Classification <- factor(ps.ls@sam_data$Classification, levels = c('H', 'PD', 'AN', 'T', 'pos', 'B'))

plot_bar(ps.ls, "Classification", "Abundance", "Phylum")+

xlab("Classification") +

ylab("Reads (counts)") +

ggtitle("Read (counts) of contaminated samples") +

geom_bar(aes(color=Phylum, fill=Phylum),

stat="identity", position='stack') +

theme_bw() +

theme(axis.title.x=element_text(size=20)) +

theme(axis.title.y=element_text(size=20)) +

theme(axis.text.x=element_text(size=20)) +

theme(axis.text.y=element_text(size=20)) +

theme(legend.text=element_text(size=20)) +

theme(legend.title=element_text(size=20))

ps.noncontam.onlybacteria@sam_data$Classification <- factor(ps.noncontam.onlybacteria@sam_data$Classification, levels = c('H', 'PD', 'AN', 'T', 'pos', 'B'))

plot_bar(ps.noncontam.onlybacteria, "Classification", "Abundance", "Phylum")+

xlab("Classification") +

ylab("Reads (counts)") +

ggtitle("Read (counts) of decontaminated samples") +

geom_bar(aes(color=Phylum, fill=Phylum),

stat="identity", position='stack') +

theme_bw() +

theme(axis.title.x=element_text(size=20)) +

theme(axis.title.y=element_text(size=20)) +

theme(axis.text.x=element_text(size=20)) +

theme(axis.text.y=element_text(size=20)) +

theme(legend.text=element_text(size=20)) +

theme(legend.title=element_text(size=20))

*##############################################################################################*

*#Exclude any samples with less than 10000 reads.*

sort(phyloseq::sample_sums(ps.noncontam.onlybacteria))

analysisdata <- subset_samples(ps.noncontam.onlybacteria, sample_sums(ps.noncontam.onlybacteria) > 10000)

analysisdata <- subset_samples(analysisdata, Classification != 'pos')

*#################################################################################################*

*#rarefaction*

analysisdatararefy = rarefy_even_depth(analysisdata, rngseed=1, sample.size = 18441, replace = FALSE, verbose = TRUE)

*##########################################################################################*

*#Build phylogenetic tree*

library(ape)

random_tree = rtree(ntaxa(analysisdatararefy), rooted=TRUE, tip.label=taxa_names(data))

physeq1 = merge_phyloseq(analysisdatararefy, random_tree)

*#####################################################################################*

*#prune asvs with less than 20 reads*

physeq2 <- prune_taxa(taxa_sums(physeq1)>=20, analysisdata)

*#Figure S8*

plot_bar(physeq2, "Classification", "Abundance", "Phylum")+

xlab("Classification") +

ylab("Reads (counts)") +

ggtitle("Read (counts) of decontaminated, pruned taxa and pruned samples") +

geom_bar(aes(color=Phylum, fill=Phylum),

stat="identity", position='stack') +

theme_bw() +

theme(axis.title.x=element_text(size=20)) +

theme(axis.title.y=element_text(size=20)) +

theme(axis.text.x=element_text(size=20)) +

theme(axis.text.y=element_text(size=20)) +

theme(legend.text=element_text(size=20)) +

theme(legend.title=element_text(size=20))

*#################################################################################################*

*#Table 1*

library(glm2)

library(stats)

donordata <- read.csv('Additional File 1 - Donor data.csv')

metadata <- read.csv("Original Data For All Groups + Focus Data with Key.xlsx - Original Data For All Groups.csv")

metadata <- subset(metadata, SampleID%in%donordata$SampleID)

donordata$Race <- as.character(metadata$Human.Race)

donordata <- subset(donordata, SampleID%in%analysisdata_pruned@sam_data$SampleID)

donordata$Race[donordata$Race == 'African American'] <- 'AFRNAMER'

donordata$Race[donordata$Race == 'WHITEOTHRACE'] <- 'White'

donordata$Race[donordata$Race == 'WHITE'] <- 'White'

donordata$Race[donordata$Race == 'Unknown'] <- 'NA'

pdvh <- subset(donordata, General.Classification%in%c("PD", "H"))

cvh <- subset(donordata, General.Classification%in%c("C", "H"))

pdvh$Age.Range<-relevel(as.factor(pdvh$Age.Range),ref="57.82")

pdvh$Menopausal.Status<-relevel(as.factor(pdvh$Menopausal.Status),ref="Post")

pdvh$BMI_Category<-relevel(as.factor(pdvh$BMI.Category),ref="Normal weight")

pdvh$History.of.Cancer<-relevel(as.factor(pdvh$History.of.Cancer),ref="No")

pdvh$Race<-relevel(as.factor(pdvh$Race),ref="White")

pdvh$General.Classification<-relevel(as.factor(pdvh$General.Classification),ref="H")

cvh$Age.Range<-relevel(as.factor(cvh$Age.Range),ref="57.82")

cvh$Menopausal.Status<-relevel(as.factor(cvh$Menopausal.Status),ref="Post")

cvh$BMI_Category<-relevel(as.factor(cvh$BMI.Category),ref="Normal weight")

cvh$History.of.Cancer<-relevel(as.factor(cvh$History.of.Cancer),ref="No")

cvh$Race<-relevel(as.factor(cvh$Race),ref="White")

cvh$General.Classification<-relevel(as.factor(cvh$General.Classification),ref="H")

#pdvh

glm <- glm(General.Classification ~ History.of.Cancer + Age.Range

+ Race + Menopausal.Status + BMI.Category,

data = pdvh, family = binomial)

summary(glm)

cvh$General.Classification <- as.factor(cvh$General.Classification)

glm <- glm(General.Classification ~ Age.Range

+ Race + Menopausal.Status + BMI.Category,

data = cvh, family = binomial)

summary(glm)

write.csv(donordata, 'Additional File 1 - Donor data (corrected).csv')

*###############################################################################*

*#Figure 2C - alpha diversity metrics - using rarefied but unpruned dataset.*

richnesschao1 <- plot_richness(physeq1, "Classification", measures=c("Observed", "Chao1", "Shannon")) +

geom_boxplot() +

theme_classic() +

xlab("") + ylab("") +

ggtitle("") +

theme(axis.title.x=element_text(size=20)) +

theme(axis.title.y=element_text(size=20)) +

theme(axis.text.x=element_text(size=20)) +

theme(axis.text.y=element_text(size=20)) +

theme(legend.text=element_text(size=20)) +

theme(legend.title=element_text(size=20))

*#Estimate richness for each metric*

results <- estimate_richness(physeq1, measures = 'Chao1')

d = sample_data(physeq1)

res <- cbind(results, d)

resultsS <- estimate_richness(physeq1, measures = 'Shannon')

res <- cbind(resultsS, res)

resultsO <- estimate_richness(physeq1, measures = 'Observed')

res <- cbind(resultsO, res)

shapiro.test(res$Chao1) *#test for normality*

*#Shapiro-Wilk normality test*

*#data: results$Chao1*

*#W = 0.75549, p-value = 5.493e-15*

model <- lm(Chao1 ~ Classification, data = res)

summary(model)

*#Estimate Std. Error t value Pr(>|t|)*

*#(Intercept) 44.7542 3.4950 12.805 <2e-16 ****

*#ClassificationPD 5.0258 7.2193 0.696 0.487*

*#ClassificationAN 3.6179 4.9427 0.732 0.465*

*#ClassificationT -0.7212 5.0227 -0.144 0.886*

shapiro.test(res$Shannon)

*#Shapiro-Wilk normality test*

*#data: res$Shannon*

*#W = 0.99361, p-value = 0.7126*

aov<- aov(Shannon~Classification, data=res)

summary(aov)

TukeyHSD(aov)

*#Tukey multiple comparisons of means*

*#95% family-wise confidence level*

*#Fit: aov(formula = Shannon ~ Classification, data = res)*

*#$Classification*

*#diff lwr upr p adj*

*#PD-H 0.12235400 -0.3333699 0.57807792 0.8979938*

*#AN-H -0.07091365 -0.3829265 0.24109918 0.9348989*

*#T-H -0.36845536 -0.6855146 -0.05139617 0.0155918*

*#AN-PD -0.19326765 -0.6489916 0.26245626 0.6892027*

*#T-PD -0.49080936 -0.9500030 -0.03161571 0.0311160*

*#T-AN -0.29754171 -0.6146009 0.01951749 0.0743863*

shapiro.test(res$Observed)

*#Shapiro-Wilk normality test*

*#data: res$Observed*

*#W = 0.76486, p-value = 1.099e-14*

model <- lm(Observed ~ Classification, data = res)

summary(model)

*#Estimate Std. Error t value Pr(>|t|)*

*#(Intercept) 44.143 3.256 13.559 <2e-16 ****

*#ClassificationPD 4.724 6.725 0.702 0.483*

*#ClassificationAN 2.265 4.604 0.492 0.623*

*#ClassificationT -1.339 4.679 -0.286 0.775*

richnesschao1$physeq1$Classification <- factor(richnesschao1$physeq1$Classification,

levels = c("H","PD", "AN", "T"))

richnessshannon <- plot_richness(physeq1, "Classification", measures=c("Shannon")) +

geom_boxplot() +

theme_classic() +

xlab("") + ylab("") +

ggtitle("") +

theme(axis.title.x=element_text(size=20)) +

theme(axis.title.y=element_text(size=20)) +

theme(axis.text.x=element_text(size=20)) +

theme(axis.text.y=element_text(size=20)) +

theme(legend.text=element_text(size=20)) +

theme(legend.title=element_text(size=20))

*#Relevel for aesthetics.*

richnessshannon$physeq1$Classification <- factor(richnessshannon$physeq1$Classification,

levels = c("H","PD", "AN", "T"))

*#Relevel for aesthetics.*

sample_data(physeq1)$Classification <- factor(sample_data(physeq1)$Classification, levels = c("H", "PD", "AN", "T"))

levels(sample_data(physeq1)$Classification)

*########################################################################################*

*#Figure 1A and B - PCoA*

*#weighted unifrac*

ord <- ordinate(physeq1, "PCoA", "UniFrac")

plot_ord <- plot_ordination(physeq1, ord, color="Classification",title="PCoA plot of Breast Tissue") +

stat_ellipse(level = 0.95) + geom_point(size=4) +

theme_bw() +

scale_color_manual(values=c("red","black","purple", "orange")) +

theme(axis.title.x=element_text(size=20)) +

theme(axis.title.y=element_text(size=20)) +

theme(axis.text.x=element_text(size=20)) +

theme(axis.text.y=element_text(size=20)) +

theme(legend.text=element_text(size=20)) +

theme(legend.title=element_text(size=20))

*#Run Betadisper to assess between sample dispersion*

*#Distance metrics.*

*#PERMANOVA and beta-dispersion functions.*

*#Distance statistics.*

distance = distance(physeq1, "UniFrac") *#add, weighted = F for unweighted stats*

groups <- sample_data(physeq1)$Classification

mod <- betadisper(distance, groups)

permutest(mod)

mod.HSD <- TukeyHSD(mod)

plot(mod.HSD)

*#p less than 0.05 suggests heterogeneity between samples - adonis not appropriate*

*#Figure S1*

*#NMDS to view dispersion among samples*

ord <- ordinate(physeq1, "NMDS", "wUniFrac") *#change to unifrac for unweighted*

> plot_ord <- plot_ordination(data, ord, color="Classification",title="PCoA plot of Breast Tissue")+

stat_ellipse(level = 0.95) + geom_point(size=4) +

theme_bw() +

scale_color_manual(values=c("red","black","purple", "orange")) +

theme(axis.title.x=element_text(size=20)) +

theme(axis.title.y=element_text(size=20)) +

theme(axis.text.x=element_text(size=20)) +

theme(axis.text.y=element_text(size=20)) +

theme(legend.text=element_text(size=20)) +

theme(legend.title=element_text(size=20))

*#################################################################################*

*#Relative abundance plots - Figure 2 D and E*

merge = merge_samples(physeq2, "Classification")

sample_data(merge)$Classification<- levels(sample_data(physeq2)$Classification)

*#Transform to relative abundance.*

merge.100 = transform_sample_counts(merge, function(x) 100 * x/sum(x))

*##plot.*

p <- plot_bar(merge.100, "SampleID", "Abundance", "Phylum") +

xlab("Classification") +

ylab("Abundance (%)") +

ggtitle("Relative Abundance Plot") +

geom_bar(aes(color=Phylum, fill=Phylum),

stat="identity", position='stack') +

theme_classic() +

theme(axis.title.x=element_text(size=20)) +

theme(axis.title.y=element_text(size=20)) +

theme(axis.text.x=element_text(size=20)) +

theme(axis.text.y=element_text(size=20)) +

theme(legend.text=element_text(size=20)) +

theme(legend.title=element_text(size=20))

*#relevel*

sample_data(merge.100)$Classification <- factor(sample_data(merge.100)$Classification, levels = c("H", "PD", "AN", "T"))

levels(sample_data(merge.100)$Classification)

*#Filtering for family level*

top100 <- names(sort(taxa_sums(physeq2), decreasing=TRUE))[1:100]

*#Transform to relative abundance.*

top100_trs <- transform_sample_counts(physeq2, function(OTU) OTU/sum(OTU))

*#Prune according to parameters set above.*

top100_prune <- prune_taxa(top100, top100_trs)

merge = merge_samples(top100_prune, "Classification")

sample_data(merge)$Classification<- levels(sample_data(physeq2)$Classification)

*#Transform to relative abundance.*

merge.100 = transform_sample_counts(merge, function(x) 100 * x/sum(x))

*##plot.*

p <- plot_bar(merge.100, "Classification", "Abundance", "Family") +

xlab("Classification") +

ylab("Abundance (%)") +

ggtitle("Relative Abundance Plot") +

geom_bar(aes(color=Family, fill=Family),

stat="identity", position='stack') +

theme_classic() +

theme(axis.title.x=element_text(size=20)) +

theme(axis.title.y=element_text(size=20)) +

theme(axis.text.x=element_text(size=20)) +

theme(axis.text.y=element_text(size=20)) +

theme(legend.text=element_text(size=20)) +

theme(legend.title=element_text(size=20))

*#relevel*

sample_data(merge.100)$Classification <- factor(sample_data(merge.100)$Classification, levels = c("H", "PD", "AN", "T"))

levels(sample_data(merge.100)$Classification)

*#Phylum level individual profiles - Figure S2*

merge.100 = transform_sample_counts(physeq2), function(x) 100 * x/sum(x))

*#Repeat the following for each group (H, PD, AN, T)*

H <- subset_samples(merge.100, Classification %in% c("H"))

p <- plot_bar(PD, "SampleID", "Abundance", "Phylum") +

xlab("SampleID") +

ylab("Abundance (%)")+

geom_bar(aes(color=Phylum, fill=Phylum),

stat="identity", position='stack') +

theme_classic() +

theme(axis.title.x=element_text(size=10)) +

theme(axis.title.y=element_text(size=10)) +

theme(axis.text.x=element_text(size=10)) +

theme(axis.text.y=element_text(size=10)) +

theme(legend.text=element_text(size=10)) +

theme(legend.title=element_text(size=10)) + theme(axis.text.x = element_text(angle = 90, vjust = -0.1, hjust=0))

p *#call plot*

*#Top 100 Family level individual profiles per tissue type - Figure S3*

top100 <- names(sort(taxa_sums(data2), decreasing=TRUE))[1:100]

top100_trs <- transform_sample_counts(data2, function(OTU) OTU/sum(OTU))

top100_prune <- prune_taxa(top100, top100_trs)

merge.100 = transform_sample_counts(top100_prune, function(x) 100 * x/sum(x))

*#repeat all steps below for each group (H, PD, AN, T)*

T <- subset_samples(merge.100, Classification %in% c("T"))

p <- plot_bar(T, "SampleID", "Abundance", "Family") +

xlab("SampleID") +

ylab("Abundance (%)")+

geom_bar(aes(color=Family, fill=Family),

stat="identity", position='stack') +

theme_classic() +

theme(axis.title.x=element_text(size=10)) +

theme(axis.title.y=element_text(size=10)) +

theme(axis.text.x=element_text(size=10)) +

theme(axis.text.y=element_text(size=10)) +

theme(legend.text=element_text(size=10)) +

theme(legend.title=element_text(size=10)) + theme(axis.text.x = element_text(angle = 90, vjust = -0.1, hjust=0))

p *#call plot*

*#################################################################################################*

*#Figure 3A*

write.csv(physeq2@tax_table, 'analysisdata_pruned_taxa_asvdesignation.csv')

*#add ASV designation, ASV1, ASV2, ASV3, etc.*

analysisdata_pruned_taxa_asvdesignation <- read.csv('analysisdata_pruned_taxa_asvdesignation.csv')

rownames(physeq2@tax_table) <- as.factor(analysisdata_pruned_taxa_asvdesignation$ASV)

colnames(physeq2@otu_table) <- as.factor(analysisdata_pruned_taxa_asvdesignation$ASV)

analysisdata_pruned_abund <- microbiome::transform(physeq2,

transform = "compositional",

target = "OTU", shift = 0,

scale = 1)

input_data <- as.data.frame(analysisdata_pruned_abund@otu_table)

input_data <- as.data.frame(t(input_data))

meta <- as.matrix(analysisdata_pruned_abund@sam_data)

meta <- as.data.frame(meta)

fit_data = Maaslin2(

input_data = input_data,

input_metadata = meta,

output = "maaslin2_output",

fixed_effects = c("Classification"),

reference = c("Classification,H"), plot_heatmap = TRUE,

min_prevalence = 0.1)

fit_data_df <- as.data.frame(fit_data$results)

fit_data_df_sig <- subset(fit_data_df, qval < 0.25)

fit_data_df <- subset(fit_data_df, fit_data_df$feature%in%fit_data_df_sig$feature)

pal <- c( "#1D91C0", "#67001F", "#CB181D", "#78C679", "#F46D43", "#A6CEE3", "#FD8D3C", "#A6D854")

maaslin2 <- ggplot(fit_data_df, aes(x=coef, y=feature, color = value, shape = value)) +

theme_classic() +xlab("Coefficient") +

geom_errorbar(aes(xmin=coef-stderr,xmax=coef+stderr), width=.2,

position=position_dodge(0.25)) +

geom_point(size=3, position=position_dodge(0.25))+

ylab("") + ggtitle("")+

xlab("") + xlab("Coefficient") + ylab("") + scale_fill_manual(values=c('black', 'black', 'black'))+ scale_color_manual(values=c('black', 'black', 'black')) +

scale_shape_manual(values=seq(0,10))+

theme(axis.title.x=element_text(size=20)) +

theme(axis.title.y=element_text(size=20)) +

theme(axis.text.x=element_text(size=20)) +

theme(axis.text.y=element_text(size=20)) +

theme(legend.text=element_text(size=20)) +

theme(legend.title=element_text(size=20))

*########################################################################################*

*#Figure 4, Raw PICRUSt2 data in Additional File 1*

pred_metagenome <- read.table('pred_metagenome_unstrat.tsv', row.names = 1, header = 1)

pred_metagenome <- t(pred_metagenome)

pred_metagenome <- make_relative(pred_metagenome)

pred_metagenome <- as.data.frame(pred_metagenome)

pred_metagenome_maaslin <- subset(pred_metagenome, rownames(pred_metagenome)%in%rownames(analysisdata_pruned@sam_data))

meta <- as.matrix(analysisdata_pruned_abund@sam_data)

meta <- as.data.frame(meta)

fit_data = Maaslin2(

input_data = pred_metagenome_maaslin,

input_metadata = meta,

output = "maaslin2_output_picrust",

fixed_effects = c("Classification"),

reference = c("Classification,H"), plot_heatmap = TRUE)

fit_data_df_picrust <- as.data.frame(fit_data$results)

*#top 50*

write.csv(fit_data_df_picrust, 'fit_data_df_picrust_all.csv')

fit_data_df_picrust_sig <- subset(fit_data_df_picrust, qval <= 3.405537e-03)

fit_data_df_picrust_sig_maaslin2 <- subset(fit_data_df_picrust, fit_data_df_picrust$feature%in%fit_data_df_picrust_sig$feature)

fit_data_df_picrust_sig_maaslin2_annotated <- read.csv('fit_data_df_picrust_sig_maaslin2_annotated_new.csv')

fit_data_df_picrust_sig_maaslin2_annotated$KEGG <- factor(fit_data_df_picrust_sig_maaslin2_annotated$KEGG, levels = c('ko:K01721 nthA; nitrile hydratase subunit alpha [EC:4.2.1.84]',

'ko:K17880 hyg; hygromycin-B 7-O-kinase [EC:2.7.1.119]',

'ko:K17744 GalDH; L-galactose dehydrogenase [EC:1.1.1.316]',

'ko:K02385 flbD; flagellar protein FlbD',

'ko:K18199 inhA; cyclohexyl-isocyanide hydratase [EC:4.2.1.103]',

'ko:K01602 rbcS, cbbS; ribulose-bisphosphate carboxylase small chain [EC:4.1.1.39]',

'ko:K00978 rfbF; glucose-1-phosphate cytidylyltransferase [EC:2.7.7.33]',

'ko:K01654 neuB, nnaB; N-acetylneuraminate synthase [EC:2.5.1.56]',

'ko:K15894 pseB, wbjB; UDP-N-acetylglucosamine 4,6-dehydratase/5-epimerase [EC:4.2.1.115 5.1.3.-]',

'ko:K19778 hdeB; acid stress chaperone HdeB',

'ko:K00772 mtaP [EC:2.4.2.28]',

'ko:K01160 rusA; crossover junction endodeoxyribonuclease RusA [EC:3.1.21.10]',

'ko:K13472 raxST; sulfotransferase',

'ko:K11731 atuD; citronellyl-CoA dehydrogenase [EC:1.3.99.-]',

'ko:K13774 atuB; citronellol/citronellal dehydrogenase',

'ko:K13777 atuF; geranyl-CoA carboxylase alpha subunit [EC:6.4.1.5]',

'ko:K16705 tuaE; teichuronic acid biosynthesis protein TuaE',

'ko:K10674 ectD; ectoine hydroxylase [EC:1.14.11.55]',

'ko:K12995 wbdC, mtfC; O-antigen biosynthesis alpha-1,3-mannosyltransferase [EC:2.4.1.348 2.4.1.-]',

'ko:K18029 nicA; nicotinate dehydrogenase subunit A [EC:1.17.2.1]',

'ko:K01768 E4.6.1.1; adenylate cyclase [EC:4.6.1.1]',

'ko:K13049 PM20D1; carboxypeptidase PM20D1 [EC:3.4.17.-]',

'ko:K00428 E1.11.1.5; cytochrome c peroxidase [EC:1.11.1.5]',

'ko:K09740 K09740; uncharacterized protein',

'ko:K09749 K09749; uncharacterized protein',

'ko:K09770 K09770; uncharacterized protein',

'ko:K09779 K09779; uncharacterized protein',

'ko:K09990 K09990; uncharacterized protein',

'ko:K08100 E1.3.3.5; bilirubin oxidase [EC:1.3.3.5]',

'ko:K05185 GABRE; gamma-aminobutyric acid receptor subunit epsilon',

'ko:K01769 E4.6.1.2; guanylate cyclase, other [EC:4.6.1.2]',

'ko:K00082 ribD2; 5-amino-6-(5-phosphoribosylamino)uracil reductase [EC:1.1.1.193]',

'ko:K01130 E3.1.6.1; arylsulfatase [EC:3.1.6.1]',

'ko:K17285 SELENBP1; methanethiol oxidase [EC:1.8.3.4]',

'ko:K03491 licR; lichenan operon transcriptional antiterminator',

'ko:K13652 K13652; AraC family transcriptional regulator',

'ko:K02012 afuA, fbpA; iron(III) transport system substrate-binding protein',

'ko:K08166 mmr; MFS transporter, DHA2 family, methylenomycin A resistance protein',

'ko:K09477 citT; citrate:succinate antiporter',

'ko:K13287 sipD, ipaD, bipD; invasin D',

'ko:K18567 pbuE; MFS transporter, DHA1 family, purine base/nucleoside efflux pump',

'ko:K19349 vga; pleuromutilin/lincosamide/streptogramin A transport system ATP-binding/permease protein',

'ko:K00680 ytmI; uncharacterized N-acetyltransferase [EC:2.3.1.-]',

'ko:K01459 E3.5.1.77; N-carbamoyl-D-amino-acid hydrolase [EC:3.5.1.77]',

'ko:K01461 E3.5.1.82; N-acyl-D-glutamate deacylase [EC:3.5.1.82]',

'ko:K02173 yggC; putative kinase',

'ko:K03830 yafP; putative acetyltransferase [EC:2.3.1.-]',

'ko:K08253 E2.7.10.2; non-specific protein-tyrosine kinase [EC:2.7.10.2]',

'ko:K10253 DODA; DOPA 4,5-dioxygenase [EC:1.14.99.-]',

'ko:K03642 rlpA; rare lipoprotein A',

'ko:K06394 spoIIIAE; stage III sporulation protein AE',

'ko:K07227 chuX; heme iron utilization protein',

'ko:K06912 tfdA; alpha-ketoglutarate-dependent 2,4-dichlorophenoxyacetate dioxygenase [EC:1.14.11.-]'

))

palletes <- c("#000000","#004949","#009292","#ff6db6","#ffb6db",

"#490092","#006ddb","#b66dff","#6db6ff","#b6dbff",

"#920000","#924900","#db6d00","#24ff24","#ffff6d","#999999",

"#E69F00", "#56B4E9", "#009E73", "#F0E442", "#0072B2", "#D55E00", "#CC79A7",

"#004949","#009292","#ff6db6","#ffb6db",

"#490092","#006ddb","#b66dff", "#888888")

maaslin2 <- ggplot(fit_data_df_picrust_sig_maaslin2_annotated, aes(x=coef, y=KEGG)) +

theme_classic() +xlab("Coefficient") +

geom_errorbar(aes(xmin=coef-stderr,xmax=coef+stderr), width=.2,

position=position_dodge(0.25)) +

geom_point(data = fit_data_df_picrust_sig_maaslin2_annotated, size=2, position=position_dodge(0.25), aes(color =General.Pathway, shape = value))+

ylab("") + ggtitle("")+

xlab("") + xlab("Coefficient") + ylab("") + scale_fill_manual(values=palletes)+ scale_color_manual(values=palletes) +

scale_shape_manual(values=seq(0,10))+

theme(axis.title.x=element_text(size=10)) +

theme(axis.title.y=element_text(size=10)) +

theme(axis.text.x=element_text(size=10)) +

theme(axis.text.y=element_text(size=10)) +

theme(legend.text=element_text(size=10)) +

theme(legend.title=element_text(size=10))

*#######################################################################################*

*# Identify DE genes with DESeq2*

library(DESeq2)

countData <- read.csv("all_samples_rawcounts.csv", row.names = 1)

metadata <- read.csv("gene_metadata.csv", row.names = 1)

dds <- DESeqDataSetFromMatrix(countData=countData, colData=metadata,

design=~Batch + Classification)

keep <- rowSums(counts(dds)) > 0

dds <- dds[keep,]

dds = DESeq(dds, test="Wald", fitType="parametric")

alpha = 0.1

sigtab = res[which(res$padj < alpha), ]

gene_list <- sigtab@rownames

normalized_counts <- counts(dds, normalized=TRUE)

subset_normalized_counts <- subset(normalized_counts, rownames(normalized_counts) %in% gene_list)

subset_normalized_counts <- as.data.frame(t(subset_normalized_counts))

pd_genes <- subset_normalized_counts[1:6,]

hc_genes <- subset_normalized_counts[7:12,]

*####################################################################################*

*#Table 2*

*# gene-asv correlations between 12 differentially abundant ASVs identified with*

*# lefse and maaslin and*

*# 48 DE genes identified with DeSeq2*

*# read in ASV relative abundance file, raw data (not relativized or decontaminated) in Additional file 1*

asv_norm <- read.csv("asv_norm.csv", header = TRUE, row.names = 1)

*# subset samples*

hc_asv <- asv_norm[1:6,]

pd_asv <- asv_norm[7:12,]

*# corr.test*

hc_asv_corr <- corr.test(hc_asv, hc_genes, method="spearman", ci=FALSE)

pd_asv_corr <- corr.test(pd_asv, pd_genes, method="spearman", ci=FALSE)

*# table of significant correlations*

hc_asv_padj <- as.data.frame(as.table(hc_asv_corr$p.adj))

hc_asv_p <- as.data.frame(as.table(hc_asv_corr$p))

hc_asv_r <- as.data.frame(as.table(hc_asv_corr$r))

hc_asv_corr_table <- cbind(hc_asv_r, hc_asv_p[,3], hc_asv_padj[,3])

*# table of significant correlations*

pd_asv_padj <- as.data.frame(as.table(pd_asv_corr$p.adj))

pd_asv_p <- as.data.frame(as.table(pd_asv_corr$p))

pd_asv_r <- as.data.frame(as.table(pd_asv_corr$r))

pd_asv_corr_table <- cbind(pd_asv_r, pd_asv_p[,3], pd_asv_padj[,3])

*#################################################################################*

*#Table 3*

*# KO-gene correlations with KO q < 0.25 and*

*# 48 DE genes identified with DeSeq2 p.adj < 0.1*

library(psych)

*#PICRUST DATA FOR RNA CORRELATION ANALYSIS - Additional file 3*

pred_metagenome <- read.table('pred_metagenome_unstrat.tsv', row.names = 1, header = 1)

pred_metagenome <- t(pred_metagenome)

pred_metagenome <- make_relative(pred_metagenome)

pred_metagenome <- as.data.frame(pred_metagenome)

pred_metagenome_rna <- subset(pred_metagenome, rownames(pred_metagenome)%in%rownames(analysisdata_pruned@sam_data))

write.table(pred_metagenome_rna, file = "pred_metagenome_samplesremoved.txt", sep = "\t",row.names = TRUE, col.names = NA)

*# read in picrust counts file transformed to relative abundance*

ko_norm <- read.csv("pred_metagenome_samplesremoved.csv", header=TRUE, sep=",", row.names=1)

*# subset out datasets*

hc_ko <- ko_norm[1:6,]

pd_ko <- ko_norm[7:12,]

*# corr.test*

hc_ko_corr <- corr.test(hc_ko, hc_genes, method="spearman", ci=FALSE)

pd_ko_corr <- corr.test(pd_ko, pd_genes, method="spearman", ci=FALSE)

*# table of significant correlations*

hc_ko_padj <- as.data.frame(as.table(hc_ko_corr$p.adj))

hc_ko_p <- as.data.frame(as.table(hc_ko_corr$p))

hc_ko_r <- as.data.frame(as.table(hc_ko_corr$r))

hc_ko_corr_table <- cbind(hc_ko_r, hc_ko_p[,3], hc_ko_padj[,3])

*# table of significant correlations*

pd_ko_padj <- as.data.frame(as.table(pd_ko_corr$p.adj))

pd_ko_p <- as.data.frame(as.table(pd_ko_corr$p))

pd_ko_r <- as.data.frame(as.table(pd_ko_corr$r))

pd_ko_corr_table <- cbind(pd_ko_r, pd_ko_p[,3], pd_ko_padj[,3])

*################################################################################*

*# scatterplots of correlations - Figures S5*

ggplot(pd_plots, aes(ASV1580,ENSG00000267676)) +

geom_point() +

theme(axis.title.x = element_text(face="italic", size=12)) +

theme(axis.title.y = element_text(face="italic", size=12)) +

labs(title="rho=1, p.adj=0") +

ylab("Gene - novel transcript") +

xlab("ASV1580: Strepococcus") +

theme(title = element_text(size=10)) +

geom_smooth(method = lm)

*##################################################################################################*

*#ASV FILES FOR LEFSE*

analysisdata_pruned_abund <- microbiome::transform(physeq2,

transform = "compositional",

target = "OTU", shift = 0,

scale = 1)

analysisdata_pruned_abund_an <- subset_samples(analysisdata_pruned_abund, Classification != "T")

analysisdata_pruned_abund_an <- subset_samples(analysisdata_pruned_abund_an, Classification != "PD")

df <- as.data.frame(analysisdata_pruned_abund_an@otu_table)

df$Classification <- as.factor(analysisdata_pruned_abund_an@sam_data$Classification)

write.table(df, file = "asv_an_lefse_file.txt", sep = "\t",row.names = TRUE, col.names = NA)

analysisdata_pruned_abund_pd <- subset_samples(analysisdata_pruned_abund, Classification != "T")

analysisdata_pruned_abund_pd <- subset_samples(analysisdata_pruned_abund_pd, Classification != "AN")

df <- as.data.frame(analysisdata_pruned_abund_pd@otu_table)

df$Classification <- as.factor(analysisdata_pruned_abund_pd@sam_data$Classification)

write.table(df, file = "asv_pd_lefse_file.txt", sep = "\t",row.names = TRUE, col.names = NA)

analysisdata_pruned_abund_t <- subset_samples(analysisdata_pruned_abund, Classification != "AN")

analysisdata_pruned_abund_t <- subset_samples(analysisdata_pruned_abund_t, Classification != "PD")

df <- as.data.frame(analysisdata_pruned_abund_t@otu_table)

df$Classification <- as.factor(analysisdata_pruned_abund_t@sam_data$Classification)

write.table(df, file = "asv_t_lefse_file.txt", sep = "\t",row.names = TRUE, col.names = NA)

*#PICRUST2 DATA FILES FOR LEFSE*

pred_metagenome <- read.table('pred_metagenome_unstrat.tsv', row.names = 1, header = 1)

pred_metagenome <- t(pred_metagenome)

pred_metagenome <- make_relative(pred_metagenome)

pred_metagenome <- as.data.frame(pred_metagenome)

analysisdata_pruned_abund_an <- subset_samples(analysisdata_pruned_abund, Classification != "T")

analysisdata_pruned_abund_an <- subset_samples(analysisdata_pruned_abund_an, Classification != "PD")

pred_metagenome_an <-subset(pred_metagenome, rownames(pred_metagenome)%in%rownames(analysisdata_pruned_abund_an@sam_data))

pred_metagenome_an$Classification <- as.factor(analysisdata_pruned_abund_an@sam_data$Classification)

write.table(pred_metagenome_an, file = "picrust2_an_lefse_file.txt", sep = "\t",row.names = TRUE, col.names = NA)

analysisdata_pruned_abund_pd <- subset_samples(analysisdata_pruned_abund, Classification != "T")

analysisdata_pruned_abund_pd <- subset_samples(analysisdata_pruned_abund_pd, Classification != "AN")

pred_metagenome_pd <-subset(pred_metagenome, rownames(pred_metagenome)%in%rownames(analysisdata_pruned_abund_pd@sam_data))

pred_metagenome_pd$Classification <- as.factor(analysisdata_pruned_abund_pd@sam_data$Classification)

write.table(pred_metagenome_pd, file = "picrust2_pd_lefse_file.txt", sep = "\t",row.names = TRUE, col.names = NA)

analysisdata_pruned_abund_t <- subset_samples(analysisdata_pruned_abund, Classification != "AN")

analysisdata_pruned_abund_t <- subset_samples(analysisdata_pruned_abund_t, Classification != "PD")

pred_metagenome_t <-subset(pred_metagenome, rownames(pred_metagenome)%in%rownames(analysisdata_pruned_abund_t@sam_data))

pred_metagenome_t$Classification <- as.factor(analysisdata_pruned_abund_t@sam_data$Classification)

write.table(pred_metagenome_t, file = "picrust2_t_lefse_file.txt", sep = "\t",row.names = TRUE, col.names = NA)

*#examples of jitterplots for taxa and genes- Figure S4*

input_data <- as.data.frame(analysisdata_pruned_abund@otu_table)

meta <- as.data.frame(analysisdata_pruned@sam_data)

input_data$Classification <- as.factor(meta$Classification)

input_data$Classification <- factor(input_data$Classification,

levels = c("H","PD", "AN", "T"))

ggplot(input_data, aes(x=Classification, y=ASV1)) +

geom_boxplot(outlier.shape=NA) + *#avoid plotting outliers twice*

geom_jitter(position=position_jitter(width=.1, height=0), pch = 1, col = "gray3") + theme_classic() +

ylab("") + ggtitle("ASV1: Staphylococcus")+

xlab("")+

theme(axis.title.x=element_text(size=10)) +

theme(axis.title.y=element_text(size=10)) +

theme(axis.text.x=element_text(size=15)) +

theme(axis.text.y=element_text(size=10)) +

theme(legend.text=element_text(size=10)) +

theme(legend.title=element_text(size=10))

*#lefse T (more abundant in H) pt. 2*

*#ASV262: Iodobacter, ASV124: Iodobacter, ASV96: Serratia*

*#ASV236: Vagococcus, ASV66: Rubrobacter, ASV449: Pseudomonas,*

*#ASV59: Brocothrix, ASV52: Acinetobacter, ASV16: Corynebacterium_1,*

*#ASV48: Iodobacter, ASV49: Pseudomonas, ASV33: Pseudomonas,*

*#ASV29: Pseudomonas, ASV21: Iodobacter, ASV24: Pseudomonas,*

*#ASV19: Pseudomonas, ASV15: Pseudomonas, ASV9: Corynebacterium_1,*

*#ASV8: Bradyrhizobium, ASV1: Staphylococcus*

*#lefse T (more abundant in H) pt. 1*

*#ASV2700: Bacteroides, ASV885: Bradyrhizobium,*

*#ASV2899: Ruminococcus_2, ASV490: Cutibacterium,*

*#ASV639: Corynebacterium_1, ASV98: Staphylococcus,*

*#ASV1034: Iodobacter, ASV535: Lactococcus, ASV503: Arthrobacter*

*#ASV987: Photobacterium, ASV484: Pseudomonas, ASV384: Pseudomonas*

*#ASV2371: Brochothrix, ASV777: Carnobacterium,*

*#ASV1591: Acinetobacter, ASV181: Iodobacter, ASV629: Pseudomonas,*

*#ASV322: Pseudomonas, ASV302: Pseudomonas, ASV227: Pseudomonas,*

*#ASV349: Pseudomonas, ASV776: Iodobacter, ASV176: Carnobacterium*

*#lefse T (more abundant in T)*

*#ASV2: Pseudomonas, ASV20: Staphylococcus, ASV41: Herbaspirillum,*

*#ASV43: Pseudogracilibacillus, ASV70: Anaerococcus, ASV87: Enterococcus,*

*#ASV42: Enterococcus, ASV190: Rhodococcus, ASV575: Pseudomonas, ASV1341: Herbaspirillum,*

*#ASV1117: Staphylococcus*

*#lefse AN (more abundant in H) pt. 2*

*#ASV124: Iodobacter, ASV96: Serratia, ASV236: Vagococcus,*

*#ASV66: Rubrobacter, ASV449: Pseudomonas, ASV59: Brochothrix,*

*#ASV52: Acetobacter, ASV16: Corynebacterium_1, ASV48: Iodobacter*

*#ASV49: Pseudomonas, ASV33: Pseudomonas, ASV29: Pseudomonas,*

*#ASV21: Iodobacter, ASV24: Pseudomonas, ASV19: Pseudomonas,*

*#ASV15: Pseudomonas, ASV9: Corynebacterium_1, ASV8: Bradyrhizobium,*

*#ASV1: Staphylococcus*

*#lefse AN (more abundant in H) pt. 1*

*#ASV2700: Bacteroides, ASV885: Bradyrhizobium,*

*#ASV2899: Ruminococcus_2, ASV490: Cutibacterium,*

*#ASV639: Corynebacterium_1, ASV98: Staphylococcus,*

*#ASV1034: Iodobacter, ASV535: Lactococcus, ASV503: Arthrobacter,*

*#ASV987: Photobacterium, ASV484: Pseudomonas,*

*#ASV384: Pseudomonas, ASV2371: Brocothrix,*

*#ASV777: Carnobacterium, ASV1591: Acinetobacter,*

*#ASV181: Iodobacter, ASV629: Pseudomonas, ASV322: Pseudomonas,*

*#ASV302: Pseudomonas, ASV227: Pseudomonas, ASV349: Pseudomonas,*

*#ASV776: Iodobacter, ASV176: Carnobacterium, ASV262: Iodobacter*

*#lefse AN (more abundant in AN)*

*#ASV2: Pseudomonas, ASV14: Atopostipes, ASV10: Oceanobacillus,*

*#ASV23: Alcaligenes, ASV27: Pseudogracilibacillus, ASV30: Duganella,*

*#ASV104: Klebsiella, ASV79: Enterococcus, ASV42: Enterococcus,*

*#ASV41: Herbaspirillum, ASV83: Tissierella, ASV68: Tissierella,*

*#ASV100: Enterococcus, ASV157: Proteus, ASV916: Oceanobacillus,*

*#ASV224: Tissierella, ASV177: Corynebacterium_1, ASV70: Anaerococcus,*

*#ASV957: Alcaligenes, ASV890: Atopostipes, ASV1341: Herbaspirillum,*

*#ASV575: Pseudomonas*

*#lefse PD*

*#ASV7: Streptococcus, ASV51: Romboutsia, ASV136: Pantoea,*

*#ASV80: Schlegelella, ASV191: Sphingomonas, ASV1580: Streptococcus,*

*#ASV1527: Romboutsia, ASV155: Sphingomonas, ASV507: Fusobacterium,*

*#ASV662: Staphylococcus, ASV415: Streptococcus, ASV16: Corynebacterium_1*

*#maaslin2*

*#asv98, asv9, asv885, asv8, asv7,*

*#asv639, asv415, asv36, asv16, asv1*

input_data <- pred_metagenome_rna

meta <- as.data.frame(analysisdata_pruned@sam_data)

input_data$Classification <- as.factor(meta$Classification)

input_data$Classification <- factor(input_data$Classification,

levels = c("H","PD", "AN", "T"))

ggplot(input_data, aes(x=Classification, y=K00799)) +

geom_boxplot(outlier.shape=NA) + *#avoid plotting outliers twice*

geom_jitter(position=position_jitter(width=.1, height=0), pch = 1, col = "gray3") + theme_classic() +

ylab("") + ggtitle("K00799")+

xlab("")+

theme(axis.title.x=element_text(size=10)) +

theme(axis.title.y=element_text(size=10)) +

theme(axis.text.x=element_text(size=15)) +

theme(axis.text.y=element_text(size=10)) +

theme(legend.text=element_text(size=10)) +

theme(legend.title=element_text(size=10))

*#LEFSE with T (more abundant in H)*

*#K03519, K01607, K01998, K07045, K01996,*

*#K01692, K03520, K07481, K11209, K01997,*

*#K01995, K16079, K01999, K11904, K07497,*

*#K00344, K01768, K00799*

*#LEFSE with T (more abundant in T)*

*#K01990, K01992, K02015, K02529, K16787, K03091*

*#K02013, K07729, K16785*

*#LEFSE with AN (more abundant in H)*

*#K03446, K03704, K01046, K00680, K03762, K07001,*

*#K00459, K01652, K02012, K01692, K00344, K07497,*

*#K07090, K00257, K00001, K01768, K07045, K08093,*

*#K07498*

*#LEFSE with AN (more abundant in AN)*

*#K06147, K02529, K01223, K03091, K01534, K02761,*

*#K02760, K02759, K07047, K00074, K07047, K00074,*

*#K03569, K02073, K03710*

*#LEFSE with PD*

*#K01915, K11073, K02012*

fit_data_df_picrust_sig_maaslin2_annotated$feature

*#"K01721" "K17880" "K17744" "K02385" "K18199"*

*#"K01602" "K00978" "K01654" "K15894" "K19778"*

*#"K00772" "K01160""K13472" "K11731"*

*#"K13774" "K13777" "K16705"*

*#"K10674" "K12995" "K18029" "K01768"*

*#"K13049" "K00428" "K09740"v"K09749"*

*#"K09770" "K09779" "K09990"*

*#"K08100" "K05185" "K01769"*

*#"K00082" "K01130" "K17285" "K03491"*

*#"K13652" "K02012" "K08166"*

*#"K09477"v"K13287" "K18567" "K19349"*

*#"K00680" "K01459" "K01461"*

*#"K02173" "K03830" "K08253" "K10253"*

*#"K03642" "K06394" "K07227"*

*#"K06912"*
